# Irradiance Independent Spectrum Reconstruction from Camera Signals Using the Interpolation Method

**DOI:** 10.3390/s22218498

**Published:** 2022-11-04

**Authors:** Yu-Che Wen, Senfar Wen, Long Hsu, Sien Chi

**Affiliations:** 1 Department of Electrophysics, National Yang Ming Chiao Tung University, No. 1001 University Road, Hsinchu 30010, Taiwan; 2 Department of Electrical Engineering, Yuan Ze University, No. 135 Yuan-Tung Road, Taoyuan 32003, Taiwan; 3 Department of Photonics, National Yang Ming Chiao Tung University, No. 1001 University Road, Hsinchu 30010, Taiwan

**Keywords:** spectrum reconstruction, spectral reflectance recovery, linear interpolation, weighted principal component analysis, multispectral imaging, camera

## Abstract

The spectrum of light captured by a camera can be reconstructed using the interpolation method. The reconstructed spectrum is a linear combination of the reference spectra, where the weighting coefficients are calculated from the signals of the pixel and the reference samples by interpolation. This method is known as the look-up table (LUT) method. It is irradiance-dependent due to the dependence of the reconstructed spectrum shape on the sample irradiance. Since the irradiance can vary in field applications, an irradiance-independent LUT (II-LUT) method is required to recover spectral reflectance. This paper proposes an II-LUT method to interpolate the spectrum in the normalized signal space. Munsell color chips irradiated with D65 were used as samples. Example cameras are a tricolor camera and a quadcolor camera. Results show that the proposed method can achieve the irradiance independent spectrum reconstruction and computation time saving at the expense of the recovered spectral reflectance error. Considering that the irradiance variation will introduce additional errors, the actual mean error using the II-LUT method might be smaller than that of the ID-LUT method. It is also shown that the proposed method outperformed the weighted principal component analysis method in both accuracy and computation speed.

## 1. Introduction

Spectral reflectance images can be used for color reproduction, medical diagnosis, and agricultural inspection [1,2,3,4,5,6,7,8,9,10]. Direct measurement using an imaging spectrometer is costly [11,12]. As an indirect measurement method, using a camera to estimate spectral reflectance is low cost [13,14,15,16]. Methods for estimating the spectral reflectance from camera signals are critical for improving measurement accuracy and detection speed. The methods can be based on basis spectra [17,18,19,20], Wiener estimation [14,21,22], regression [23,24,25,26,27,28], and interpolation [29,30,31,32,33,34]. The basis-spectrum methods assume that the target spectrum to be reconstructed is a linear combination of the basis spectra derived from training samples. The weighting coefficients for the basis spectra are solved from simultaneous equations describing camera signals. Regression and Wiener estimation methods build a transformation matrix from training samples to the convert the low-dimensional camera signals to the high-dimensional spectral reflectance. The interpolation method uses the neighboring reference spectra in camera signal space to interpolate the target spectrum. Since the basis spectra and transformation matrix are derived from all training samples, the estimation accuracy using the interpolation method can be higher than the other three methods.

For example, the authors of [29,30,31,32,33,34] showed that the interpolation method can be more accurate than two basis-spectrum methods, principal component analysis (PCA) [17,18], and nonnegative matrix transformation [19,20]. The interpolation method can still be more accurate even compared to the enhanced basis-spectrum methods [30,31,32,33,34]. The enhanced methods use the basis spectra that emphasize the relationship between the target and training samples at the expense of computation time [18,20]. Due to the use of a look-up table (LUT) to store the reference spectra, the interpolation method is often called the LUT method [29]. The LUT method is computationally two orders of magnitude faster than the enhanced basis-spectrum methods [31,33].

Using the LUT method, a simplex mesh in signal space is built from reference samples. The simplex enclosing the target sample is located. The reference samples of the simplex vertices are used to interpolate the target sample. If the target sample is outside the convex hull of the reference samples in signal space, it cannot be interpolated and must be extrapolated instead. The target sample is called an outside sample to distinguish it from the samples inside the convex hull [30,31,32,33,34]. The extrapolation problem limits the usability of the LUT method. The authors of [33] proposed the auxiliary reference samples (ARSs) to extrapolate the outside samples. The results showed that the extrapolation error utilizing the ARSs is lower than other extrapolation methods in [31,32].

The spectral reflectance image is reconstructed pixel-by-pixel using the methods in [17,18,19,20,21,22,23,24,25,26,27,29,30,31,32,33,34], i.e., the spectrum of a pixel is reconstructed from the camera signals of the pixel. For example, the authors of [32] showed spectral reflectance images reconstructed using the LUT method. The regression method using a deep-learning neural network can reconstruct spectral reflectance images taking into account the spatial structure of the image [28]. This approach is attractive, although the reconstructed spectra are shown to be less accurate in color [26].

Indirect measurement methods using cameras share common limitations compared to direct measurement methods using imaging spectrometers. (1) Illuminant-dependent training/reference samples are required. An optimal set of training/reference samples for one set of test samples may not be optimal for another. Therefore, field applications require proper selection of the training/reference samples [35]. One of the most suitable applications for indirect methods is the recovery of spectral reflectance in industrial products and artworks, where the spectral properties of the illuminant and pigments are known. (2) The accuracy of the reconstructed spectra is limited by the number of camera channels.

**Figure 1:**
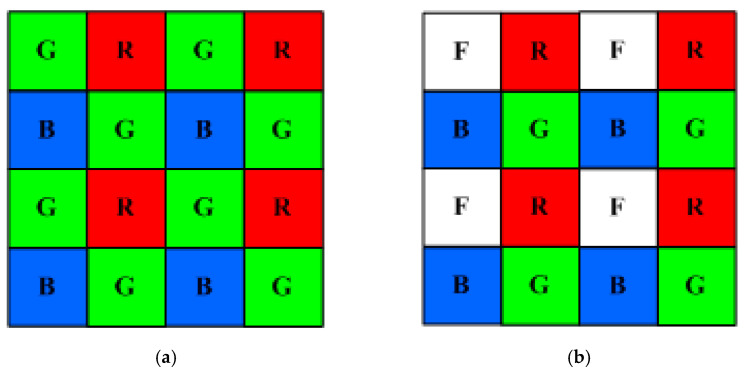
Schematic diagrams showing (**a**) Bayer color filter array and (**b**) modified Bayer color filter array.

A conventional tricolor camera has three channels available, where the Bayer color filter array (CFA) is used to improve the spatial resolution, as shown in Figure 1a. One unit cell of the Bayer CFA includes one red, two green, and one blue square filters. Since the accuracy of the reconstructed spectrum increases with the number of signal channels, the use of a quadcolor camera and the LUT method to improve the estimation accuracy was investigated [34]. The CFA of the quadcolor camera is compatible with the Bayer CFA, as shown in Figure 1b, and the demosaicing algorithm also can be applied. In Figure 1b, one green filter on the Bayer CFA unit cell is modified as the white square. The quadcolor camera was found to be effective in improving the estimation accuracy, even when the fourth channel did not use a color filter. However, for the quadcolor camera, the computation time using the LUT method is approximately doubled compared to the weighted PCA (wPCA) method, although the mean spectrum reconstruction error is smaller [34]. The wPCA method is an enhanced basis-spectrum method [18]. The LUT method is time-consuming for the quadcolor camera because locating a simplex in 4D signal space is computationally two orders of magnitude slower than locating a simplex in 3D signal space.

A spectrum reconstruction method is irradiance-independent if the irradiance on the target sample is multiplied by a factor, and the spectral power density of the reconstructed spectrum is multiplied by the same factor. Interpolation in the signal space is irradiance-dependent, since the reconstructed spectrum shape depends on the sample irradiance. The irradiance-dependent LUT (ID-LUT) method is suitable for reconstructing the spectrum under the condition that the target sample and the reference samples have the same irradiance [29,30,31,32,33,34]. In field applications, the illuminant brightness may vary over time and the irradiance varies with the distance between the target sample and the illuminant. Therefore, an irradiance-independent LUT (II-LUT) method is needed. Basis-spectrum methods can be irradiance-independent but must equivalently use one signal channel to represent the sample irradiance. Since the number of signal channels is equivalently reduced by one, using an irradiance-independent basis-spectrum method, the error in reconstructing the spectrum shape of the target sample may increase compared to the corresponding irradiance-dependent method.

This paper proposes an II-LUT method for spectrum reconstruction. This method interpolates the spectrum shape and luminance of the target sample in the normalized signal space using the normalized reference spectra. A tricolor and a quadcolor camera were taken as example cameras. Since the normalized signal space is 3D, the computation time of the II-LUT method for the quadcolor camera is slightly longer than that of the ID-LUT method in 3D for the tricolor camera. Reference and test samples prepared using the Munsell color chips were used as examples. The illuminant was D65. It was found that for the considered quadcolor camera, the mean recovered spectral reflectance error of the test samples using the II-LUT method was slightly larger than that of the test samples using the ID-LUT method without considering the irradiance variation. Therefore, using the proposed method has the advantages of irradiance independence and computation time saving, but at the cost of the increase in the mean error. The results were compared to the irradiance-dependent wPCA (ID-wPCA) and irradiance-independent wPCA (II-wPCA) methods.

The organization of this paper is as follows. Section 2.1, Section 2.2, Section 2.3 describe the considered camera spectral sensitivities, color samples, and the assessment metrics for the recovered spectral reflectance, respectively. Section 3.1, Section 3.2, Section 3.3, Section 3.4 describe the ID-wPCA, II-wPCA, ID-LUT, and II-LUT methods, respectively. Section 3.5 briefly introduces the extrapolation method for the ID-LUT and II-LUT methods. Section 4.1 shows the effect of the irradiance variation on the spectrum reconstruction using the ID-LUT and ID-wPCA methods. Section 4.2 shows the numerical results using the II-LUT and II-wPCA methods. A camera color device model (CDM) converts camera signals to tristimulus values. Section 4.3 compares the II-LUT method with the irradiance-independent color device model (II-CDM) [36] to predict the tristimulus values from the camera signals. Section 5 gives the conclusions. For ease of reference, the Abbreviations section lists the abbreviations defined herein in alphabetical order.

## 2. Materials and Assessment Metrics

### 2.1. Camera Spectral Sensitivities

In this paper, spectra were sampled from 400–700 nm in step of 10 nm. The spectrum is represented by the vector ***S*** = [*S*(400 nm), *S*(410 nm), …, *S*(700 nm) ]^T^, where *S*(*λ*) is the spectral amplitude at wavelength *λ*; and the subscript T denotes the transpose operation. The number of sampling wavelengths *M*_w_ = 31.

The Nikon D5100 and RGBF cameras considered in [34] were taken as the tricolor and quadcolor camera examples, respectively. The red (R), green (G), and blue (B) channels of the RGBF camera were assumed to be the same as the Nikon D5100 camera, their spectral sensitivity vectors are designated as ***S***_CamR_, ***S***_CamG_, and ***S***_CamB_, respectively [37]. The spectral sensitivity vector of the fourth channel of the RGBF camera is designated as ***S***_CamF_ and was assumed to be the product of the spectral sensitivity of a typical silicon sensor [38] and the spectral transmittance of a Baader UV/IR cut filter. The fourth channel is a greenish yellow channel and is designated as the F channel due to being free of color filter.

A color filter can be applied to the fourth channel to modify its spectral sensitivity. However, for simplicity, the RGBF camera without the color filter was considered. Taking the RGBF camera as the quadcolor camera example does not lose the generality of the proposed irradiance independent method.

### 2.2. Color Samples

The color samples were taken the same as in [33,34]. Samples were prepared using reflectance spectra of matt Munsell color chips measured with a spectroradiometer [39], where 1268 reflectance spectra were used. Illuminant D65 was assumed to be the light source. The same 202 and 1066 color chips in [33,34] were chosen for preparing the reference/training and test samples, respectively. The reference samples for the LUT methods and the training samples for the wPCA methods were the same.

The reflection spectrum vector from a color chip can be calculated as

(1) $$S_{\mathrm{Reflection}}=S_{\mathrm{Ref}}\circ S_{D65},$$

where ***S***_Ref_ and ***S***_D65_ are the spectral reflectance vector of the color chip and the spectrum vector of the illuminant D65, respectively; and the operator ∘ is the element-wise product. The maximum spectral power density of the spectrum ***S***_D65_ used to prepare reference samples and test samples were assumed to be 1 and *S_Max_*, respectively.

*S_Max_* = 1 + Δ*I*_Test_, (2)

where Δ*I*_Test_ is the deviation of the irradiance. The value of Δ*I*_Test_ was set to be zero unless otherwise specified. The color points of light reflected from the 1268 Munsell color chips in the CIELAB color space have been shown in [33]. This paper adopted the CIE 1931 color matching functions.

**Figure 2:**
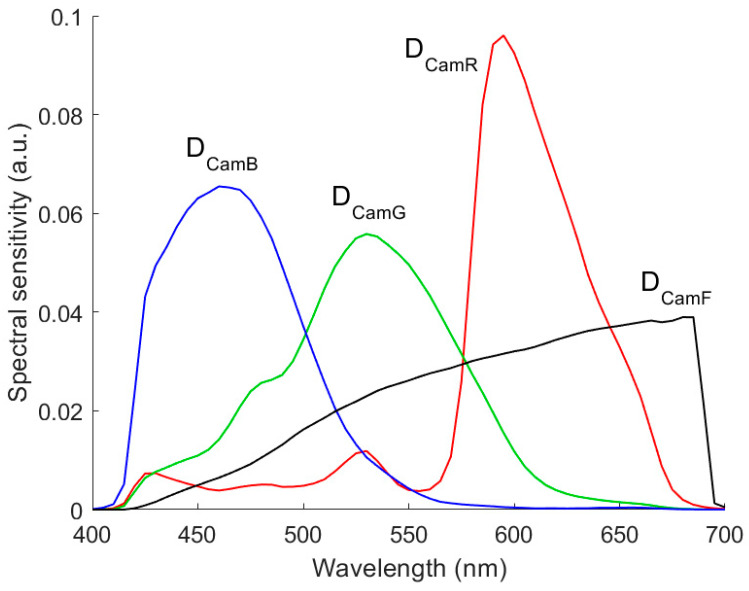
The spectral sensitivities of red (***D***_CamR_), green (***D***_CamG_), blue (***D***_CamB_), and F (***D***_CamF_) channels of the RGBF camera under the white balance condition.

Under the white balance condition, the spectral sensitivity vector and signal value of a signal channel of the RGBF camera are ***D***_Cam*U*_ = ***S***_Cam*U*_/(***S***_White_∘***S***_D65_)^T^***S***_Cam*U*_ and *U* = ***S***_Reflection_^T^***D***_Cam*U*_, respectively, for *U* = *R*, *G*, *B*, and *F*; and ***S***_White_ is the spectral reflectance vector of a white card. The same white card in [28,29] was taken, which is the white side of a Kodak gray card. Figure 2 shows the vectors ***D***_CamR_, ***D***_CamG_, ***D***_CamB_, and ***D***_CamF_. The vector representing the camera signals is designated as ***C*** = [*R*, *G*, *B*]^T^ and [*R*, *G*, *B*, *F*]^T^ for the D5100 and RGBF cameras, respectively. The color points of reflection spectra from Munsell color chips in the RGB and RGBF signal spaces have been shown in [33,34], respectively.

### 2.3. Assessment Metrics

For a given test signal vector, the methods to reconstruct the reflection light spectrum are shown in Section 3. The spectral reflectance vector ***S***_RefRec_ can be calculated as the reconstructed reflection spectrum vector ***S***_Rec_ divided by the D65 spectrum vector ***S***_D65_ element by element. The same metrics in [33,34] were used to assessment the reconstructed results. They are briefly described below. The root mean square (RMS) error *E*_Ref_ and goodness-of-fit coefficient GFC were used to assess the reconstructed spectral reflectance vector ***S***_RefRec_, where *E*_Ref_ *=* (|***S***_RefRec_ – ***S***_Ref_|^2^/*M*_w_)^1/2^, GFC = |***S***_RefRec_^T^***S***_Ref_|/|***S***_RefRec_| |***S***_Ref_| and |·| stands for the norm operation. CIEDE2000 Δ*E*_00_ was used to assess the color difference between ***S***_Rec_ and ***S***_Reflection_. The spectral comparison index (SCI), which represents an index of metamerism, was also used to assess the reconstructed results [40], where the parameter *k* = 1 in the formula for calculating SCI shown in [40].

The values of *E*_Ref_, Δ*E*_00_ and SCI are the smaller the better. The mean *μ*, standard deviation *σ*, 50th percentile PC50, 98th percentile PC98, and maximum MAX of the three metrics were calculated. The value of GFC is the larger the better. The mean *μ*, standard deviation *σ*, 50th percentile PC50, and minimum MIN of the metric GFC were calculated. If GFC > 0.99, the spectral curve shape is well fitted [32,41]. The ratio of samples with GFC > 0.99 was calculated. This ratio of good fit is designated as RGF99.

## 3. Methods

The details of the ID-LUT and ID-wPCA methods have been described in [33,34]. The following sections briefly describe them in order to compare them with II-wPCA and II-LUT methods. The ID-LUT and II-LUT methods are described by taking the RGBF camera as an example.

### 3.1. The ID-wPCA Method

The training samples were used to derive the principal components using the PCA method [42]. The signal vector of the test sample was assumed to be

(3) $$C=C_{0}+\sum_{k=1}^{N_{C}}d_{k}Q_{k},$$

where ***C***_0_ = ***D***_Cam_^T^***P***_0_; *N*_C_ is the number of camera channels; ***Q****_k_* = ***D***_Cam_^T^***P****_k_*; ***D***_Cam_ is the camera’s spectral sensitivity matrix with dimension *M*_w_x*N*_C_; ***P***_0_ is the average spectrum vector of the training samples; *d_k_* and ***P****_k_* are the coefficient and spectrum vector of the *k*-th principal component, respectively. For the RGBF camera, *N*_C_ = 4 and ***D***_Cam_ = [***D***_CamR_ ***D***_CamG_ ***D***_CamB_ ***D***_CamF_]. Given the spectral sensitivity matrix and the training samples, the coefficient *d_k_*, *k* = 1, 2, …, *N*_C_, can be solved from Equation (3). The reconstructed reflection spectrum vector is

(4) $$S_{\mathrm{Rec}}=P_{0}+\sum_{k=1}^{N_{C}}d_{k}P_{k}.$$

The first three and four principal components were used as the basis spectra to reconstruct the spectrum for the tricolor and quadcolor cameras, respectively. If the reconstructed spectrum has negative values, the value is set to zero.

The wPCA method is the same as the PCA method shown above, except that the training samples were weighted according to the sample to be reconstructed [18,34]. A training sample was multiplied by a weighting factor Δ*E_i_* ^−*γ*^, where Δ*E_i_* is the color difference between the test sample and the *i*-th training sample in CIELAB color space; and *γ* is a constant. Basis spectra were derived from the weighted training samples. If *γ* =0, the wPCA method becomes the PCA method. The value of *γ* is usually set to 1.0 [18,33]. The value of *γ* was optimized for the minimum mean *E*_Ref_ of the test samples for individual camera in this paper. The third-order root polynomial regression model (RPRM) was used to convert signal values to tristimulus values to calculate Δ*E* [36]. This model was trained using the training samples.

### 3.2. The II-wPCA Method

The II-wPCA method is the same as the ID-wPCA method, except that the signal vector and reconstructed reflection spectrum vector are

(5) $$C=d_{0}C_{0}+\sum_{k=1}^{N_{C}-1}d_{k}Q_{k},$$

(6) $$S_{\mathrm{Rec}}=d_{0}P_{0}+\sum_{k=1}^{N_{C}-1}d_{k}P_{k}.$$

Compared to Equations (3) and (4), in Equations (5) and (6), ***C***_0_ and ***P***_0_ are multiplied by the coefficient *d*_0_; and the upper limit of the summation index is modified from *N*_C_ to *N*_C_ –1 so that the coefficients *d*_0_ and *d_k_*, *k* = 1, 2, …, *N*_C_ –1, can be solved from Equation (5).

The reconstruction spectrum can be written as Equation (4), since it was assumed that the irradiance of the training samples and test samples are the same. If the irradiance of the test sample is different from the training samples, the coefficient *d*_0_ needs to be used to represent the change in the irradiance.

### 3.3. The ID-LUT Method

Reference samples were used to generate the simplex mesh. Simplices are triangles and tetrahedra in 2D and 3D signal spaces, respectively. The simplex in 4D signal space is beyond imagination. The number of vertices of a simplex is *N*_C_ +1. The simplex enclosing the vector ***C*** in the signal space was located. The signal vector of the test sample was assumed to be

(7) $$C=\sum_{k=1}^{N_{C}+1}\alpha_{k}C_{k},$$

where ***C****_k_* is the *k*-th signal vectors of the reference samples at the vertices of the simplex enclosing the signal vector ***C*** in the signal space; the coefficient *α_k_* is the barycentric coordinate describing the location of the color point in the simplex and

(8) $$1=\sum_{k=1}^{N_{C}+1}\alpha_{k}.$$

If the color point of ***C*** is inside the simplex, 0 < *α_k_* < 1. The coefficients *α_k_*, *k* = 1, 2, …, *N*_C_ +1, were solved from Equations (7) and (8). The reconstructed reflection spectrum vector is

(9) $$S_{\mathrm{Rec}}=\sum_{k=1}^{N_{C}+1}\;\alpha_{k}S_{k},$$

where ***S****_k_* is the reference spectrum vector corresponding to the *k*-th vertex.

If a signal vector is outside the convex hull of the simplex mesh, it cannot be interpolated and must be extrapolated. The convex hull of reference samples cannot be plotted due to its 4D geometry for the RGBF camera. The number of outside samples is 340. These outside samples in the RGB, GBF, BFR, and FRG signal space were shown in [34]. The extrapolation of the outside samples is described in Section 3.5.

For the case that the irradiance is increased by a factor of *κ*, the signal vector ***C*** in Equation (7) becomes *κ**C***. However, due to the constraint Equation (8), the reconstructed spectrum ***S***_Rec_ in Equation (9) does not become *κ**S***_Rec_. In addition, since the location of the test sample in the signal space varies with the factor *κ*, the simplex enclosing ***C*** may vary accordingly. Therefore, this interpolation method is irradiance-dependent.

### 3.4. The II-LUT Method

The II-LUT method is similar to the ID-LUT method. The test sample was interpolated in the normalized signal space, where a signal component is normalized to the sum of all signal components. For example, the normalized signals for the RGBF camera are

*r = R/(R + G + B + F)*, (10)

*g = G/(R + G + B + F)*, (11)

*b = B/(R + G + B + F)*, (12)

The normalized signal vector is defined as ***c*** = [*r*, *g*, *b*]^T^. Normalized reference samples were used to generate the simplex mesh. The simplex is a tetrahedron for the RGBF camera because the dimension of the normalized signal vector is one less than the signal vector.

The simplex enclosing the vector ***c*** in the normalized signal space was located. The number of vertices of the simplex is *N*_C_. The normalized signal vector of the test sample was assumed to be

(13) $$c=\sum_{k=1}^{N_{C}}\beta_{k}c_{k},$$

where ***c****_k_* is the *k*-th signal vector of the normalized reference samples at the vertices of the simplex enclosing the signal vector ***c*** in the normalized signal space; the coefficient *β_k_* is the barycentric coordinate describing the location of the color point in the simplex and

(14) $$1=\sum_{k=1}^{N_{C}}\beta_{k}.$$

If the color point of ***c*** is inside the simplex, 0 < *β_k_* < 1. The coefficients *β_k_*, *k* = 1, 2, …, *N*_C_, were solved from Equations (13) and (14).

The normalized reconstructed reflection spectrum vector is

(15) $$N_{\mathrm{Rec}}=\sum_{k=1}^{N_{C}}\beta_{k}N_{k},$$

where ***N****_k_* is the normalized reference spectrum vector corresponding to the *k*-th vertex and

***N****_k_* = ***S****_k_*/***S****_k_*^T^***D***_CamT_, (16)

where ***S****_k_* is the *k*-th reference spectrum ***S****_k_* for *k* = 1, 2, …, *N*_C_; and ***D***_CamT_ = ***D***_CamR_ + ***D***_CamG_ + ***D***_CamB_ + ***D***_CamF_. Note that ***S****_k_*^T^***D***_CamT_ = *R_k_* + *G_k_* + *B_k_* + *F_k_*, where *R_k_*, *G_k_*, *B_k_*, and *F_k_* are the signal values of the *k*-th vertex. The same normalization factor was used for the signal values and spectra. The reconstructed spectrum is

***S***_Rec_ = *Y**N***_Rec_/***N***_Rec_^T^***u****_y_* , (17)

where ***u****_y_* = [$\bar{y}$(400 nm),$\bar{y}$(410 nm), …,$\bar{y}$(700 nm)]^T^ is the vector representing the color matching function $\bar{y}$; *Y* is the interpolated Y stimulus value, calculated as

(18) $$Y=\sum_{k=1}^{N_{C}}\eta_{k}\beta_{k}Y_{k},$$

*η_k_ =* (*R*+*G*+*B*+*F*)/ (*R_k_*+*G_k_*+*B_k_*+*F_k_*), (19)

and *Y_k_* is the Y stimulus value of the *k*-th vertex.

For the case where the irradiance is multiplied by a factor, the corresponding color coordinate vector ***c*** remains unchanged, and the spectral shape of test sample ***N***_Rec_ in Equation (15) also remains unchanged. Therefore, this interpolation method is irradiance-independent.

Substituting Equations (15), (16) and (18) into Equation (17), the reconstruction spectrum can be written as

(20) $$S_{\mathrm{Rec}}=\sum_{k=1}^{N_{C}}\eta_{k}\beta_{k}S_{k}.$$

**Figure 3:**
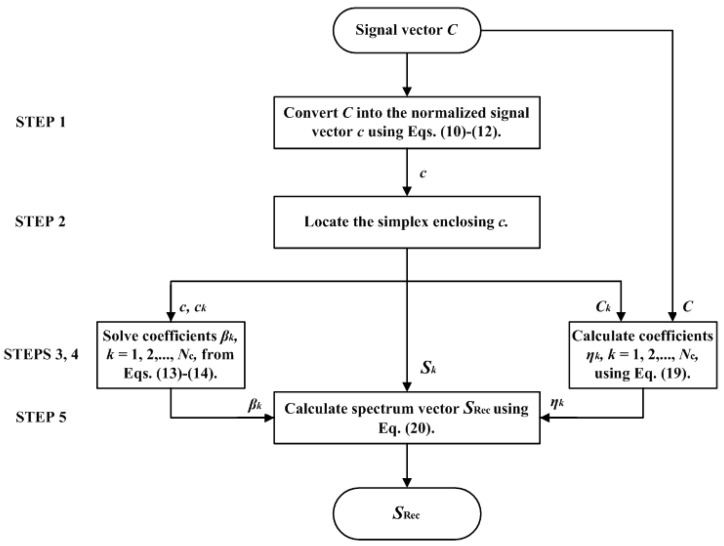
Flow chart to interpolate the test sample using the II-LUT method.

In summary, there are five steps to interpolate the test sample using the II-LUT method, where the flow chart is shown in Figure 3.

STEP 1: Convert the signal vector ***C*** of the test sample into the normalized signal vector ***c***.
STEP 2: Locate the simplex enclosing the vector ***c*** in the normalized signal space.
STEP 3: Solve the coefficients *β_k_*, *k* = 1, 2, …, *N*_C_, from Equations (13) and (14).
STEP 4: Calculate the coefficients *η_k_*, *k* = 1, 2, …, *N*_C_, according to Equation (19).
STEP 5: Calculate the reconstruction spectrum ***S***_Rec_ according to Equation (20).

From either Equation (13) or Equation (20), the following equation can be derived

(21) $$C=\;\;\sum_{k=1}^{N_{C}}\eta_{k}\beta_{k}C_{k},$$

where ***C*** and ***C****_k_* are the signal vectors corresponding to ***c*** and ***c****_k_*, respectively. It seems that Equations (21) and (20) are similar to Equations (7) and (9), respectively, but the vertices are chosen in a different way, the number of reference spectra is reduced by one, and the reference spectra are weighted differently.

**Figure 4:**
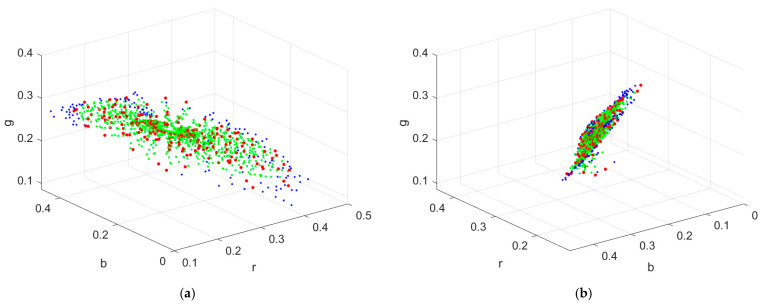
(**a**) Color points of the reference samples, inside samples, and outside samples in the rgb normalized signal space with red, green, and blue dots, respectively, where the camera is the RGBF camera. (**b**) The same as (**a**) except for viewing angle.

**Figure 5:**
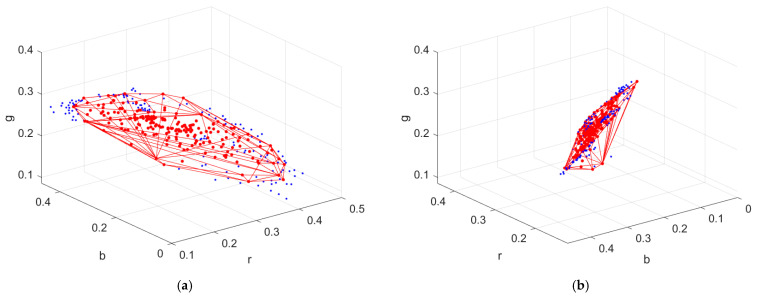
(**a**) The convex hull of the reference samples in the rgb normalized signal space for the RGBF camera. Reference and outside samples are shown with red and blue dots, respectively. (**b**) The same as (**a**) except for viewing angle.

Figure 4a shows the color points of the reference samples, inside samples and outside samples in the rgb normalized signal space for the RGBF camera with red, green, and blue dots, respectively. Figure 4b is the same as Figure 4a except for viewing angle. The convex hull of the reference samples is shown in Figure 5a,b, where the viewing angles are the same as those in Figure 4a,b, respectively. As can be seen from Figure 4a,b, most of the reference and test samples lie almost in a plane. Therefore, the shape of some tetrahedra generated from the reference samples is “thin”. The solutions to the coefficients in Equations (13) and (14) are unique. Since the coefficients are the barycentric coordinates describing the location of a point in the tetrahedron, each coefficient varies between 0 and 1 even for a “thin” tetrahedron.

If a signal vector is outside the convex hull of the tetrahedral mesh, it cannot be interpolated and must be extrapolated. The number of outside samples for the RGBF camera is 131, which are shown with blue dots in Figure 5a,b. The method to extrapolate outside samples is described in Section 3.5. Since the samples were projected from the 4D signal space to the 3D signal space, the number of outside samples was reduced from 340 using the ID-LUT method to 131 using the II-LUT method.

### 3.5. Extrapolation

The outside samples of the ID-LUT and II-LUT methods were extrapolated using the reference samples and ARSs. ARSs are measured using appropriately selected color filters and color chips so that they are highly saturated. The color filters are called the ARS filters. The extrapolation process is the same as the interpolation method shown in Section 3.3 and Section 3.4 but using the expanded reference samples including the ARSs. The same cyan, magenta, yellow, red, green, and blue ARS filters and reference color chips as in [34] were adopted to extrapolate the cases using the D5100 and RGBF cameras. All outside samples can be extrapolated using the ID-LUT and II-LUT methods utilizing the reference samples and ARSs.

**Figure 6:**
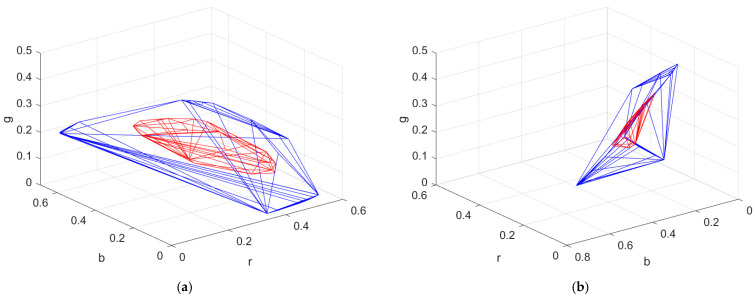
(**a**) The convex hull of the ARSs in the rgb normalized signal space for the RGBF camera. The convex hull of the reference samples is shown in red for comparison. (**b**) The same as (**a**) except for viewing angle.

The convex hull of the ARSs in RGBF signal space cannot be plotted due to its 4D geometry. The number of the ARSs in the convex hull is 126. The color points of these ARSs in the RGB, GBF, BFR, and FRG signal space were shown in [34]. Figure 6a,b show the convex hull of the ARSs in the RGB normalized signal space at different viewing angles. The black sample is not used as an ARS for extrapolation using the II-LUT method. The number of the ARSs in the convex hull is 30. The convex hull of the reference samples in the RGB normalized signal space is also shown in red in Figure 6a,b for comparison. We can see that the convex hull of the ARSs is expanded compared to the convex hull of the reference samples.

## 4. Results and Discussion

### 4.1. Irradiance Dependent Spectrum Reconstruction

**Figure 7:**
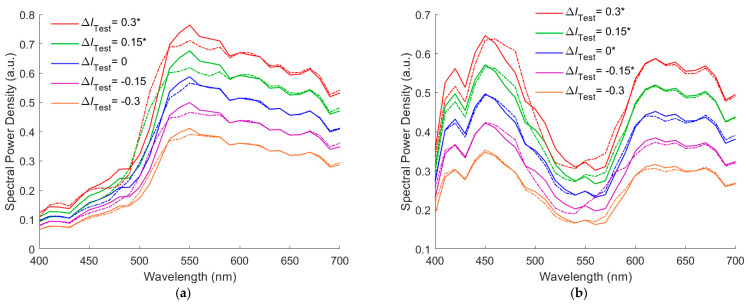
(**a**) Target spectrum ***S***_Reflection_ (solid line) and the reconstructed spectra ***S***_Rec_ (dashed line) for the cases with the irradiance deviation Δ*I*_Test_ = −0.3, −0.15, 0, 0.15, and 0.3, where the RGBF camera and the ID-LUT method are used. The test sample is prepared using the 5Y 8.5/8 color chip. (**b**) The same as (**a**) except that the color chip is 10P 7/8. Outside sample are indicated with “*” in the legend.

To show the effect of the irradiance variation on the spectrum reconstruction using the RGBF camera and the ID-LUT method, Figure 7a shows the target spectra of test samples *S*_Reflection_ and the reconstructed spectra *S*_Rec_ for the cases with the irradiance deviation Δ*I*_Test_ = −0.3, −0.15, 0, 0.15, 0.3. In Figure 7a, the color chip used for preparing the test sample is 5Y 8.5/8 in Munsell annotation; outside samples are indicated with “*”. Figure 7b is the same as Figure 7a, except that the color chip is 10P 7/8. The test samples in Figure 7a,b are inside sample and outside sample, respectively, when Δ*I*_Test_ = 0. As the Δ*I*_Test_ value changes, an inside sample may become an outside sample, and vice versa. From Figure 7a,b , it can be seen that the amplitude of the reconstructed spectra *S*_Rec_ increases with the irradiance deviation Δ*I*_Test_, but the spectrum shape changes with the irradiance deviation Δ*I*_Test_. The effect of the irradiance variation on the spectrum reconstruction can be clearly seen from Figure 7a,b.

**Figure 8:**
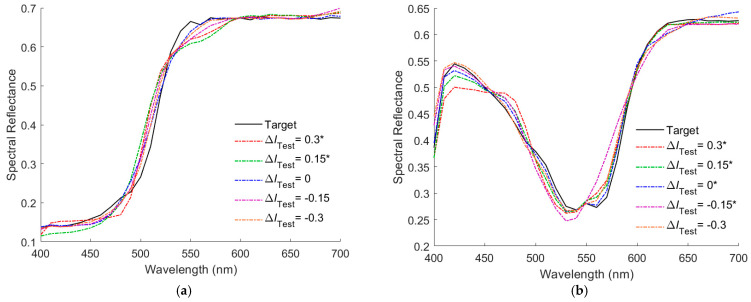
(**a**,**b**) showing the target reflectance spectra and the recovered reflectance spectra for the cases in Figure 7a,b, respectively. Outside samples are indicated with “*” in the legend.

Figure 8a,b show the recovered spectral reflectance *S*_RefRec_ for the cases shown in Figure 7a,b, respectively, where the illuminant spectrum vector of the correct amplitude was used to calculate the *S*_RefRec_ from the *S*_Rec_. Outside samples are indicated with “*” in the figures. In Figure 8a, the RMS error *E*_Ref_ = 0.0165, 0.0237, 0.0175, 0.0350, and 0.0301 for the cases of Δ*I*_Test_ = −0.3, −0.15, 0, 0.15, 0.3, respectively. In Figure 8b, *E*_Ref_ = 0.0171, 0.0282, 0.0108, 0.0141, and 0.0229 for the cases of Δ*I*_Test_ = −0.3, −0.15, 0, 0.15, 0.3, respectively.

**Figure 9:**
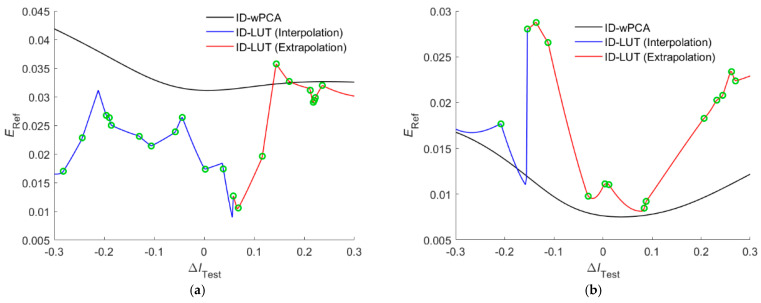
RMS error *E*_RefRec_ value versus the Δ*I*_Test_ value using the ID-LUT and ID-wPCA methods. The camera is the RGBF camera. The test samples are prepared using the (**a**) 5Y 8.5/8 and (**b**) 10P 7/8 color chips. Green circles show the change of the located simplex as the Δ*I*_Test_ value increases.

Figure 9a,b show the *E*_Ref_ value versus the Δ*I*_Test_ value for the test samples prepared with the color chips 5Y 8.5/8 and 10P 7/8, respectively. The two color chips were used in Figure 8a,b. For the case in Figure 9a, when Δ*I*_Test_ > 0.058, the test sample became the outside sample. For the case in Figure 9b, when Δ*I*_Test_ < −0.154, the test sample became the inside sample. Figure 9a,b show the zigzag variation of the *E*_Ref_ value. Around a turn point in the figure, different simplices in RGBF signal space were used for interpolation/extrapolation. The green circles in Figure 9a,b represent the change of the located simplex as the Δ*I*_Test_ value increases. Figure 9a,b also show the cases using the ID-wPCA method with the optimized *γ* = 1.2. Using the ID-wPCA method, the *E*_Ref_ value varies continuously with the Δ*I*_Test_ value.

The ID-LUT method can be used to accurately recover spectral reflectance without irradiance deviation (Δ*I*_Test_ =0). However, due to its irradiance dependence, the ID-LUT method may not be suitable for field applications where the irradiance of the test sample differs from that of the reference sample, as shown above.

### 4.2. Irradiance Independent Spectrum Reconstruction

Section 4.2.1 and Section 4.2.2 show the results using the RGBF camera and the II-LUT method. Section 4.2.3 shows the results using the RGBF camera and the II-wPCA method. Section 4.2.4 shows the results for the D5100 camera using the II-LUT and II-wPCA methods. The deviation Δ*I*_Test_ = 0 was assumed, since the Δ*I*_Test_ value does not affect the spectral shape reconstructed using the II-LUT and II-wPCA methods. If the examples shown in Figure 7a,b are reconstructed using the II-LUT method; the reconstructed spectra are identical except for the amplitude. Although the spectral shape of the recovered reflectance is also independent of the Δ*I*_Test_ value, its amplitude depends on the amplitude of the illuminant spectrum vector. The issue of measuring/estimating the amplitude of the illuminant spectrum vector is beyond the scope of this paper. Irradiance on 2D objects can be easily measured/estimated, but not on 3D objects. However, at least the relative spectral reflectance can be reconstructed using the II-LUT method, which can be useful if properly calibrated. In this section, the recovered spectral reflectance was calculated using the illuminant spectrum vector of the correct amplitude.

Spectrum reconstruction using the ID-LUT and ID-wPCA methods for the RGBF and D5100 cameras have been considered in [34], where the issue of irradiance deviation was not considered, i.e., Δ*I*_Test_ = 0 was assumed. The spectrum reconstruction results using the ID-LUT and ID-wPCA method shown in [34] are given below for comparison. However, for the results shown, the reconstruction error using the ID-LUT and ID-wPCA method may increase in practice due to the irradiance deviation, as shown in Section 4.1.

#### 4.2.1. Using the RGBF Camera and the II-LUT Method: Examples of Reconstructed Spectra

**Figure 10:**
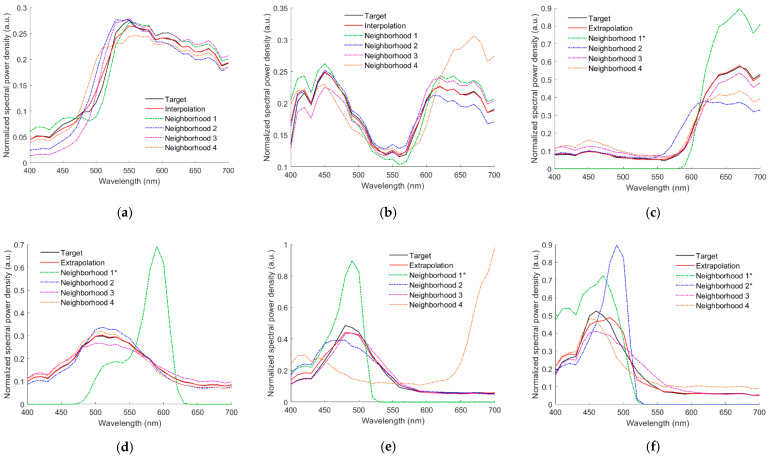
Normalized target spectrum ***N***_Reflection_, reconstructed spectra ***N***_Rec_, and neighboring reference spectra using the RGBF camera and II-LUT method. Munsell annotations of the color chips are (**a**) 5Y 8.5/8, (**b**) 10P 7/8, (**c**) 2.5R 4/12, (**d**) 2.5G 7/6, (**e**) 10BG 4/8, and (**f**) 5PB 4/12, respectively. Normalized ARS neighborhood is indicated with “*” in the legend.

Figure 10a–d show the normalized reconstructed spectra ***N***_Rec_ of the light reflected from the 5Y 8.5/8, 10P 7/8, 2.5R 4/12, 2.5G 7/6, 10BG 4/8, and 5PB 4/12 color chips, respectively, using the RGBF camera and the II-LUT method. In Figure 10a–d, the normalized target spectrum ***N***_Reflection_ and neighboring reference spectra are also shown, where ***N***_Reflection_ = ***S***_Reflection_ / (*R* + *G* + *B* + *F*) according to the same normalization definition in Equation (16) and the reflection spectrum ***S***_Reflection_ is defined in Equation (1). The color chips 5Y 8.5/8 and 10P 7/8 are the same as those used in Figure 8a,b, respectively. The cases in Figure 10a,b are interpolation examples. The cases in Figure 10c–f are extrapolation examples. For the cases in Figure 10c–f, the number of referenced ARSs is 1, 1, 1, and 2, respectively. The normalized ARS neighborhood is indicated with “*” in the figures. Except for the case in Figure 10f, the spectra were reconstructed well. For the cases shown in Figure 10a–f, Table 1 shows the values of the coefficient *β_k_* defined in Equation (13), where the maximum *β_k_* value is shown in bold and the *β_k_* value corresponding to the normalized ARS neighborhood is indicated with “*”. For the cases in Figure 10c–f, the main contribution to the normalized reconstructed spectra ***N***_Rec_ comes from the normalized reference sample. However, for the cases in Figure 10c,f, the contribution of the normalized ARS neighborhood to the ***N***_Rec_ is not negligible because of its larger *β_k_* value as shown in Table 1.

**Figure 11:**
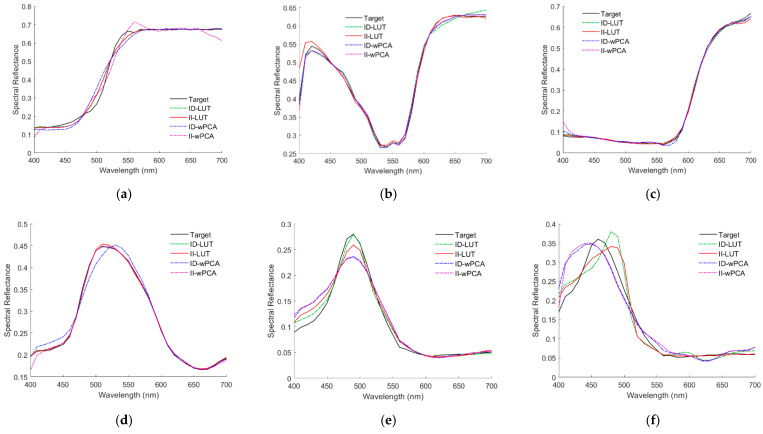
(**a**–**f**) showing the target spectra ***S***_Ref_ and recovered reflectance spectra ***S***_RefRec_ for the cases in Figure 10a,b, respectively. The spectrum reconstruction methods are shown in figures.

The recovered spectral reflectance ***S***_RefRec_ for the cases in Figure 10a–f are shown in Figure 11a–f, respectively, which also show the cases using the ID-LUT method for comparison. Table 2 shows the *E*_Ref_ and Δ*E*_00_ values for the cases shown in Figure 11a–f. For the cases shown, the spectral reflectance can be better recovered using the ID-LUT method except for the cases in Figure 11c,f. However, the reconstruction error using the ID-LUT method may increase due to the irradiance deviation. Figure 9a,b show the error increase for the cases shown in Figure 11a,b, respectively.

#### 4.2.2. Using the RGBF Camera and the II-LUT Method: Assessment Metric Statistics

Table 3 shows the assessment metric statistics for the test samples using the ID-LUT and II-LUT methods with the RGBF camera. The number of outside samples is 340 and 131 for the ID-LUT and II-LUT methods, respectively. The reduction in the number of outside samples using the II-LUT method is due to the projection of color samples from 4D RGBF space to 3D rgb space. It can be seen from Table 3 that the mean *E*_Ref_ values using the ID-LUT method for all samples, inside samples, and outside samples were 0.0089, 0.0077, and 0.0115, respectively; the mean *E*_Ref_ values using the II-LUT method for all samples, inside samples, and outside samples were 0.0093, 0.0089, and 0.0119, respectively. The mean error of the outside samples was larger than that of the inside samples. Compared to the ID-LUT method, the mean *E*_Ref_ values using the II-LUT method for all samples, inside samples, and outside samples were increased by 4.2%, 16.2%, and 2.8%, respectively. Note that when using the II-LUT method, 340 − 131 = 209 outside samples in RGBF space became inside samples in rgb space. Therefore, the error increase ratio was higher for the inside samples.

The increase in the mean *E*_Ref_ of the test samples was slight. The mean GFC values of test samples using the ID-LUT and II-LUT methods were almost the same. Surprisingly, the mean Δ*E*_00_ and SCI values of test samples using the II-LUT were reduced compared to the ID-LUT method. The improvement in the mean Δ*E*_00_ and SCI values using the II-LUT method is due to the smaller color difference error of the inside samples.

**Figure 12:**
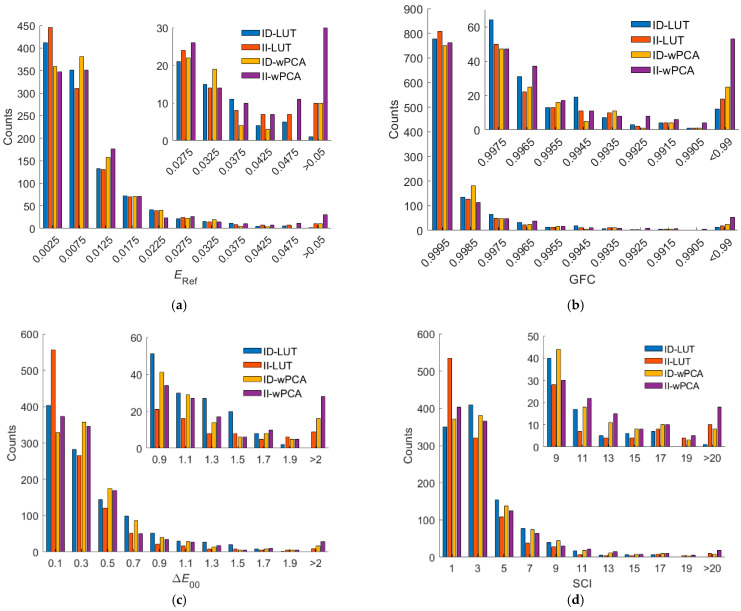
(**a**) *E*_Ref_, (**b**) GFC, (**c**) Δ*E*_00_, and (**d**) SCI histograms for the 1066 test samples. The camera is the RGBF camera. The spectrum reconstruction methods are shown in figures. The insets show enlarged parts.

Figure 12a–d show the *E*_Ref_, GFC, Δ*E*_00_, and SCI histograms for the test samples, respectively, where the ID-LUT and II-LUT methods were used. It can be seen from the figures that for bins with small *E*_Ref_, Δ*E*_00_, and SCI values, the number of counts using the II-LUT method was higher than that using the ID-LUT method. For bins with large *E*_Ref_, Δ*E*_00_, and SCI values, the number of counts using the II-LUT method was also higher than that using the ID-LUT method. On average, the performance using the ID-LUT method and II-LUT method was about the same.

#### 4.2.3. Using the RGBF Camera and the II-wPCA Method

For the RGBF camera, the spectral reflectance S_RefRec_ recovered from the light reflected from the color chips in Figure 10a–f using the ID-wPCA and II-wPCA methods are also shown in Figure 11a–f, respectively. The optimized γ = 1.2 and 1.4 for the ID-wPCA and II-wPCA methods, respectively. Table 2 also shows the E_Ref_ and ΔE_00_ values for the cases using the ID-wPCA and II-wPCA methods. For the cases shown, the spectral reflectance can be recovered better using the II-LUT method compared to the II-wPCA method, except for the case in Figure 11b. From Table 2, it can be seen that in the cases of Figure 11a,b,d, the spectral reflectance can be better recovered using the II-wPCA method compared to the ID-wPCA method, although the basis spectra is one less. However, the performance assessment of the method should refer to the statistical results.

Table 4 shows the assessment metric statistics for the test samples using the ID-wPCA and II-wPCA methods with the RGBF camera. The inside and outside samples using the wPCA methods are the same as the corresponding LUT methods, although the wPCA methods can be used to reconstruct all test samples. Since the number of basis spectra is one less, the mean *E*_Rec_ of the test samples using the II-wPCA method is larger than the ID-wPCA method. It can be seen from Table 4 that the mean *E*_Ref_ values using the ID-wPCA method for all samples, inside samples, and outside samples were 0.0095, 0.0080, and 0.0127, respectively; the mean *E*_Ref_ values using the II-wPCA method for all samples, inside samples and outside samples were 0.0129, 0.0119, and 0.020, respectively. The mean error of the outside samples is larger than the inside samples due to extrapolation [33,34]. Compared to the ID-wPCA method, the mean *E*_Ref_ values using the II-wPCA method for all samples, inside samples and outside samples were increased by 36.2%, 49.3%, and 58.1%, respectively.

As can be seen from Table 4, the ratio of good fit RGF99 for the outside samples decreased from 0.9382 using the ID-wPCA method to 0.855 using the II-wPCA method. Using the II-wPCA method, 14.5% of the outside samples had GFC values less than 0.99. The maximum *E*_Ref_, Δ*E*_00_ and SCI values were significantly increased using the II-wPCA method compared to the ID-wPCA method. Figure 12a–d also show the assessment metric histograms using the ID-wPCA and II-wPCA methods with the RGBF camera. The results show that the performance using the II-wPCA was severely degraded compared to the ID-wPCA method. However, the spectrum reconstruction error using the ID-wPCA method may increase due to the irradiance deviation, as shown for the examples in Figure 9a,b.

As can be seen from Table 3 and Table 4, for all assessment metric statistics, the II-LUT method outperformed the II-wPCA method. Compared to the II-wPCA method, the mean *E*_Ref_ values of test samples using the II-LUT method was reduced by 28.0%. The PC98 and MAX values of *E*_Ref_ using the II-LUT method were significantly smaller than those using the II-wPCA method. The ratio of good fit RGF99 for the outside samples using the II-LUT method remained high.

#### 4.2.4. Using the D5100 Camera

Table 5 shows the assessment metric statistics for the test samples using the ID-LUT and II-LUT methods with the D5100 camera. The II-LUT method for the RGB camera is the same as for the RGBF camera shown in Section 3.4, except that the dimension of signal space is reduced from 3 to 2 and the normalized signals are *r* = *R*/(*R*+*G*+*B*) and *g* = *G*/(*R*+*G*+*B*). The number of outside samples is 202 and 63 for the ID-LUT and II-LUT methods, respectively.

It can be seen from Table 5 that the mean *E*_Ref_ values using the ID-LUT method for all samples, inside samples, and outside samples were 0.0131, 0.0120, and 0.0180, respectively; the mean *E*_Ref_ values using the II-LUT method for all samples, inside samples, and outside samples were 0.0179, 0.0170, and 0.0314, respectively. Compared to the ID-LUT method, the mean *E*_Ref_ values using the II-LUT method for all samples, inside samples, and outside samples were increased by 36.3%, 42.2%, and 74.6%, respectively. The ratio of good fit RGF99 for the outside samples decreased from 0.9208 using the ID-LUT method to 0.8413 using the II-LUT method. Using the II-LUT method, 15.87% of the outside samples had GFC values less than 0.99. As shown in Section 4.2.2, the mean spectrum reconstruction error using the ID-LUT and II-LUT methods was about the same for the RGBF camera. Compared to the ID-LUT method for the D5100 camera, the mean spectrum reconstruction error using II-LUT was significantly increased. For the case with the D5100 camera and the II-LUT method, the use of two normalized signals was not sufficient to reconstruct the spectra well. Using the II-LUT method, the mean *E*_Ref_ with the D5100 camera was approximately double that with the RGBF camera.

Table 6 shows the assessment metric statistics for the test samples using the ID-wPCA and II-wPCA methods with the D5100 camera. The optimized *γ* = 1.7 and 1.6 for the ID-wPCA and II-wPCA methods, respectively. The inside and outside samples using the wPCA methods are the same as the corresponding LUT methods. As expected, the mean *E*_Rec_ value of the test samples using the II-wPCA method was larger than that using the ID-wPCA method.

It can be seen from Table 6 that the mean *E*_Ref_ values using the ID-wPCA method for all samples, inside samples, and outside samples were 0.0121, 0.0110, and 0.0169, respectively; the mean *E*_Ref_ values using the II-wPCA method for all samples, inside samples, and outside samples were 0.0186, 0.0164, and 0.0527, respectively. Compared to the ID-wPCA method, the mean *E*_Ref_ values using the II-wPCA method for all samples, inside samples, and outside samples were increased by 53.76%, 49.95%, and 212.2%, respectively. The ratio of good fit RGF99 for the outside samples decreased from 0.8713 using the ID-wPCA method to 0.6349 using the II-wPCA method. Using the II-wPCA method, 36.51% of the outside samples had GFC values less than 0.99. Compared to the ID-wPCA method, the maximum *E*_Ref_, Δ*E*_00_ and SCI values were significantly increased using the II-wPCA method. For the case with the D5100 camera and the II-wPCA method, the use of two basis spectra was not sufficient to reconstruct the spectra well.

**Figure 13:**
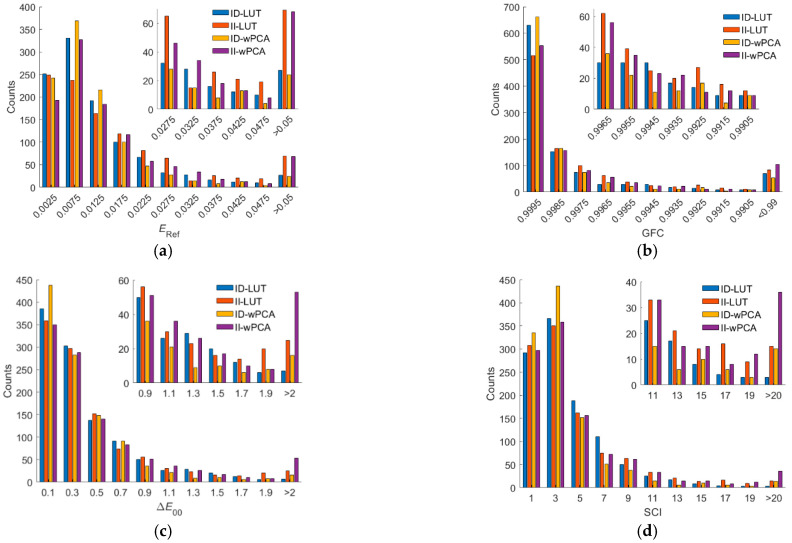
(**a**) *E*_Ref_, (**b**) GFC, (**c**) Δ*E*_00_, and (**d**) SCI histograms for the 1066 test samples. The camera is the D5100 camera. The corresponding spectrum reconstruction methods are shown in figures. The insets show enlarged parts.

Figure 13a–d show the assessment metric histograms using the ID-LUT, II-LUT, ID-wPCA, and II-wPCA methods with the D5100 camera. For bins with large *E*_Ref_, Δ*E*_00_, and SCI values, the number of counts using the irradiance independent method (II-LUT or II-wPCA method) method is much higher than that using the irradiance dependent method (ID-LUT or ID-wPCA method). The results show that the performance using the irradiance independent method was severely degraded compared to the irradiance dependent method.

#### 4.2.5. Computation Time Comparison

All programs in this paper were implemented in MATLAB (version R2021a, MathWorks). The MATLAB functions “delaunay” and “pointLocation” were used to generate the simplex mesh and locate the simplex in 2D or 3D space, respectively [33]. The MATLAB functions “delaunayn” and “tsearchn” were used to generate the simplex mesh and locate the simplex in 4D space, respectively, because the MATLAB functions “delaunay” and “pointLocation” do not support dimensions greater than 3 [34]. On the Windows 10 platform, the computation time required to reconstruct the spectral reflectance vector ***S***_RefRec_ from the signal vector ***C*** using the D5100 camera and the ID-LUT method is taken as the reference unit time.

Using the D5100 camera, the ratio of the computation time required to use the ID-LUT method, the II-LUT method, the ID-wPCA method, and the II-wPCA method was 1: 1.33: 51.6: 51.4. Using the II-LUT method takes a longer time than the ID-LUT method. In [33], the ratio of the computation time required to use the ID-LUT method and the ID-wPCA method was 1: 80.2. Due to the improvement of the program code of the wPCA method, the computation speed using the wPCA method in this work is faster than our previous work in [33].

Using the RGBF camera, the ratio of the computation time required to use the ID-LUT method, the II-LUT method, the ID-wPCA method, and the II-wPCA method was 108.9: 1.52: 53.7: 53.3. The computation speed using the II-LUT method was 108.9/1.52 = 71.6 times faster than the ID-LUT method. The computation speed using the II-LUT method was 53.3/1.52 = 35.1 times faster than the II-wPCA method.

### 4.3. Irradiance Independent Color Device Model (II-CDM)

In the wPCA methods, RPRM was used to convert the camera signals to the tristimulus values for calculating the weighting factors. RPRM is a popular II-CDM in which the converted tristimulus values increase linearly with irradiance [36]. The Δ*E*_00_ statistics presented in Table 3, Table 4, Table 5 and Table 6 show the performance of predicting the tristimulus values for the test samples using the II-LUT and II-wPCA methods. The II-LUT and II-wPCA methods can also be used as II-CDMs, although they require more computation time compared to RPRM. The II-wPCA method is not suitable as II-CDM because the computation is time-consuming and the PC98 and MAX values of Δ*E*_00_ are large, as shown in Table 4 and Table 6. However, it is interesting to compare the Δ*E*_00_ statistics using the II-LUT method and RPRM.

Since the reconstructed spectrum using the II-LUT method can be written as Equation (20), the tristimulus vector *T* = [*X*, *Y*, *Z*]^T^ of the test sample can be written as

(22) $$T\;=\;\sum_{k=1}^{N_{C}}\eta_{k}\beta_{k}T_{k},$$

where *T_k_* is tristimulus vector of the *k*-th reference sample. Equation (18) is the stimulus Y component of Equation (22). The II-LUT method can be modified to II-CDM by Equation (22), called II-LUT-CDM, as shown below. The procedure to convert the camera signals to the tristimulus values is the same as the five-step procedure of the II-LUT method shown in Section 3.4 except for the STEP 5. STEP 5 of II-LUT-CDM uses Equation (22) to calculates the tristimulus values. Using II-LUT-CDM, Table 3 and Table 5 show the Δ*E*_00_ statistics for the RGBF and D5100 cameras, respectively; Figure 12c and Figure 13c show the Δ*E*_00_ histograms for the RGBF and D5100 cameras, respectively.

**Figure 14:**
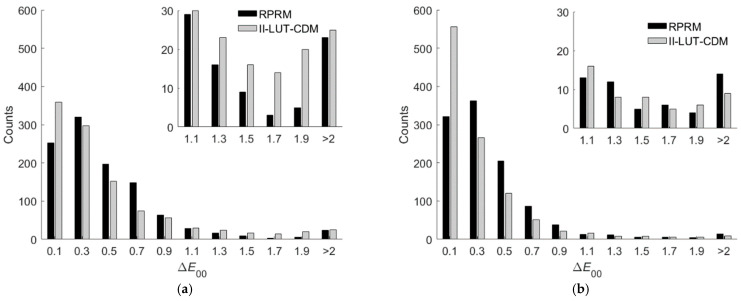
The Δ*E*_00_ histograms for the 1066 test samples using the (**a**) D5100 camera and (**b**) RGBF camera. The corresponding camera device models are shown in figures. The insets show enlarged parts.

Table 7 shows the Δ*E*_00_ statistics for test samples using the third-order RPRM. In Table 7, the cases using the D5100 and RGBF cameras are shown, where the RGB and RGBF signal values were used as variables for regression, respectively. For both cases, the optimized root polynomial order is 3. The mean Δ*E*_00_ value using the RGBF camera is smaller than the mean using the D5100 camera. Using RPRM, Figure 14a,b show the Δ*E*_00_ histograms for the RGBF and D5100 cameras, respectively, where the results using II-LUT-CDM are also shown for comparison.

As can be seen from the Δ*E*_00_ statistics shown in Table 3, Table 5, and Table 7, the prediction accuracy was improved using the RGBF camera compared to the D5100 camera. For the D5100 camera, the prediction accuracy using II-LUT-CDM was slightly better than RPRM. For the RGBF camera, the prediction accuracy using II-LUT-CDM was significantly better than RPRM. For the “Δ*E*_00_ = 0.1” bins shown in Figure 14a,b, the number of counts using II-LUT-CDM is larger than that using RPRM. For the “Δ*E*_00_ > 2” bin shown in Figure 14b, the number of counts using II-LUT-CDM is smaller than that using RPRM.

Note from Table 7 that using RPRM, the PC98 and MAX values of Δ*E*_00_ for the outside samples are very large. The test sample is called an outside sample because it cannot be enclosed using the training samples in the signal space, as shown in [33,34]. Therefore, using RPRM to predict the tristimulus values of the outside samples is extrapolation. Extrapolation using polynomial regression is notoriously unreliable, as is the root polynomial regression. It was found that if the ARSs were included in the training samples of RPRM, the prediction accuracy was not better.

### 4.4. Reconstruction of Spectral Reflectance Images

The II-LUT method was used to reconstruct spectral reflectance images using the RGBF camera. Test images were prepared using multispectral images from the open-source CAVE dataset representing the spectral reflectance of the materials in the scene [43]. Image values for the CAVE dataset are 16-bit unsigned integers computed from multispectral images measured with a tunable filter. The value of a pixel varies with the actual irradiance on the pixel. Since the CAVE dataset does not provide pixel irradiance, test images were prepared assuming a maximum spectral reflectance of 0.9 and uniform illumination using the illuminant D65. The value of 0.9 is approximately the maximum spectral reflectance of the Munsell color chips. The reference/training samples were the same as in the previous sections.

**Figure 15:**
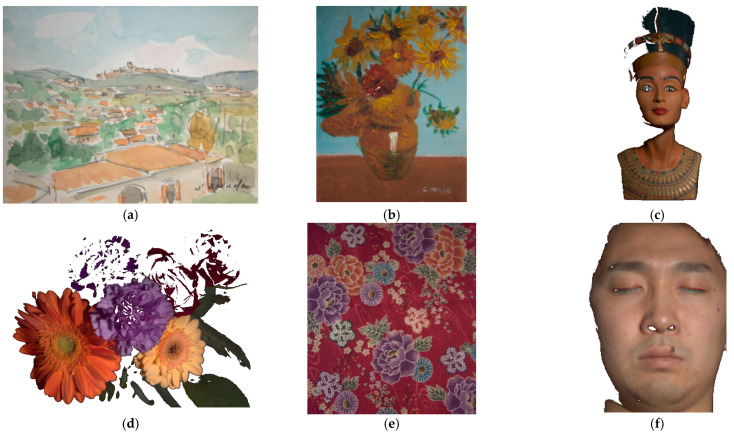
Test images: (**a**) “watercolors”, (**b**) “oil painting”, (**c**) “egyptian statue”, (**d**) “flowers”, (**e**) “cloth”, and (**f**) “face”. Nearly black pixels were removed. The white dots near the center of (**b**) indicate red-yellow pixels that cannot be extrapolated using the II-LUT method.

Figure 15a–f show six test images cropped from the CAVE sample images to remove the background, which are (a) “watercolors”, (b) “oil painting”, (c) “egyptian statue”, (d) “flowers”, (e) “cloth”, and (f) “face”. The cropped image resolutions of Figure 15a–f are (a) 483 × 375, (b) 369 × 483, (c) 204 × 439, (d) 483 × 381, (e) 449 × 511, and (f) 218 × 300. Nearly black pixels were removed from the test images due to the low signal-to-noise ratio (SNR) at the time of measurement and the low spectral reflectance accuracy in the CAVE dataset. Most of the removed pixels are black backgrounds and shadows.

Table 8 shows the assessment metric statistics for the spectral reflectance images reconstructed from the test images in Figure 15a–f using the II-LUT method and RGBF camera, where the number of image pixels considered are shown. A total of 66 red-yellow pixels near the center of Figure 15b (“oil painting”) cannot be extrapolated due to high saturation, which are shown in white dots and were not included in the statistics in Table 8. These 66 pixels could be extrapolated utilizing the appropriate additional ARSs. Table 9 is the same as Table 8 except that the II-wPCA method was used. Since the reference/training samples were not prepared to meet the characteristics of the materials in the scene, the assessment metric statistics shown in Table 8 and Table 9 are worse than those shown in Table 3 and Table 4, respectively.

Compared with the mean *E*_Ref_ value of 0.0093 in Table 3, using the II-LUT method, the mean *E*_Ref_ values for the “watercolors”, “oil painting”, “egyptian statue”, “flowers”, “cloth” and “face” images are 1.99, 1.65, 0.97, 2.02, 2.55, and 2.63 times, respectively. The 202 Munsell color chips used to prepare the reference/training samples lack spectral reflectance samples for skin. Note that some pixels on the forehead, nose, and cheeks have reflected glare as shown in Figure 15f. The spectral reflectance recovery of such pixels is poor.

The mean *E*_Ref_ values for the “oil painting”, “egyptian statue”, “flowers”, and “cloth” images using the II-wPCA method are roughly the same as using the II-LUT method. The mean *E*_Ref_ value for the “watercolors” image using the II-wPCA is significantly larger than using the II-LUT method. The mean *E*_Ref_ value for the “face” image using the II-wPCA is significantly smaller than using the II-LUT method. Note that the maximum values of the *E*_Ref_, Δ*E*_00_, and *SCI* metrics are significantly large when using the II-wPCA method, as shown in Table 4 and Table 9. For the II-wPCA method, the value of the coefficient *d_k_* solved from Equation (5) can be very large, while for the II-LUT method, the value of the coefficient *β_k_* solved from Equations (13) and (14) is restricted to be between 0 and 1.

In Table 8 and Table 9, better mean metric values using the II-LUT method compared to the II-wPCA method are shown in bold and vice versa. Using the II-LUT method compared to the II-wPCA method, the mean *E*_Ref_ values for the “watercolors” and “cloth” images are smaller; the mean *GFC*, Δ*E*_00_, and *SCI* values for the “watercolors”, “flowers”, and “cloth” images are better.

**Figure 16:**
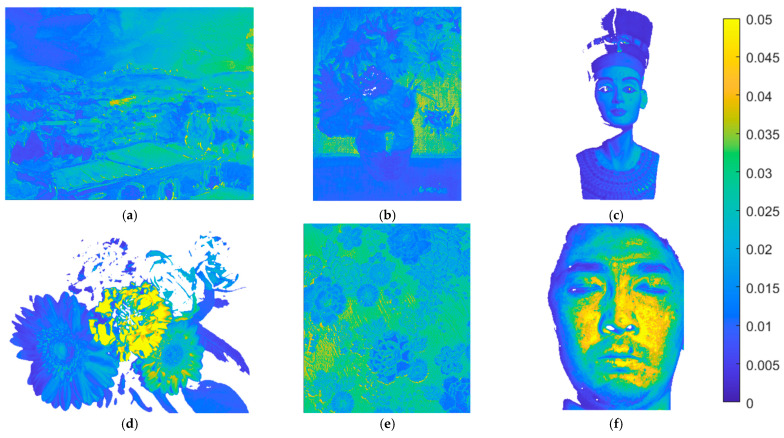
(**a**–**f**) showing the *E*_Ref_ maps for the test images in Figure 15a–f, respectively, using the II-LUT method and RGBF camera. Pixel *E*_Ref_ values greater than 0.05 are set to 0.05.

**Figure 17:**
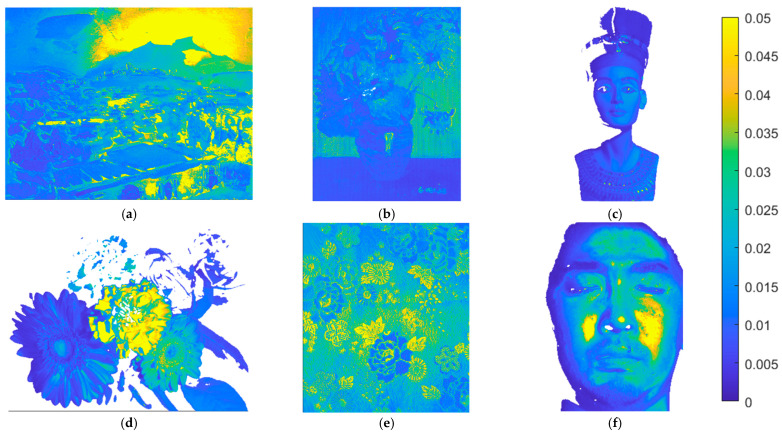
(**a**–**f**) showing the *E*_Ref_ maps for the test images in Figure 15a–f, respectively, using the II-wPCA method and RGBF camera. Pixel *E*_Ref_ values greater than 0.05 are set to 0.05.

The metric map of the test images is used to show the spatial distribution of the assessment metric values. Using the II-LUT method and RGBF camera, Figure 16a–f show the *E*_Ref_ maps for the test images in Figure 15a–f, respectively. Pixel *E*_Ref_ values greater than 0.05 are set to 0.05. Figure 17a–f are the same as Figure 16a–f, respectively, except that the II-wPCA method was used. For the *E*_Ref_ maps of the “watercolors” and “cloth” images, using the II-LUT method is significantly better than using the II-wPCA method. For the *E*_Ref_ map of the “face” image, using the II-LUT method is worse than using the II-wPCA method.

**Figure 18:**
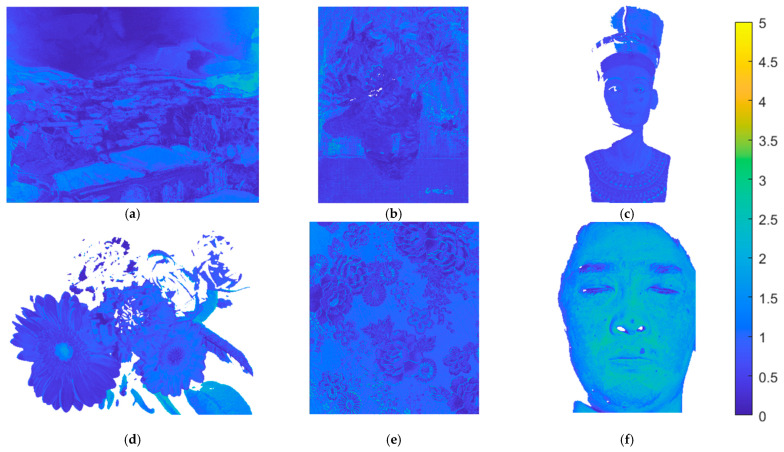
(**a**–**f**) showing the Δ*E*_00_ maps for the test images in Figure 15a–f, respectively, using the II-LUT method and RGBF camera. Pixel Δ*E*_00_ values greater than 5.0 are set to 5.0.

**Figure 19:**
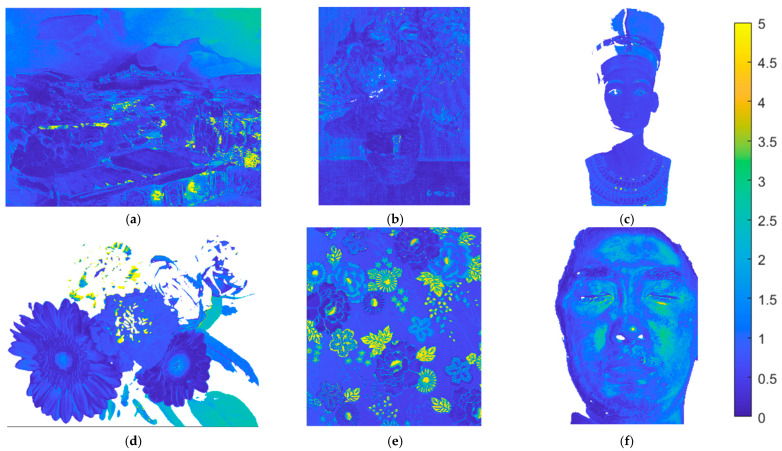
(**a**–**f**) showing the Δ*E*_00_ maps for the test images in Figure 15a–f, respectively, using the II-wPCA method and RGBF camera. Pixel Δ*E*_00_ values greater than 5.0 are set to 5.0.

Using the II-LUT method and RGBF camera, Figure 18a–f show the Δ*E*_00_ maps for the test images in Figure 15a–f, respectively. Pixel Δ*E*_00_ values greater than 5.0 are set to 5.0. Figure 19a–f are the same as Figure 18a–f, respectively, except that the II-wPCA method was used. For the Δ*E*_00_ maps of the “watercolors” and “cloth” images, using the II-LUT method is significantly better than using the II-wPCA method. For the Δ*E*_00_ map of the “face” image, using the II-LUT method is worse than using the II-wPCA method.

The above results show that, as expected, the selection of reference/training samples is crucial for the spectrum reconstruction using the II-LUT and II-wPCA methods. The selection of the 202 reference samples was not optimized for the test images. However, the mean spectrum reconstruction error using the II-LUT method was low or moderate for most image pixels, except for the “face” image and the purple flower for the “flowers” image. Using the II-LUT method avoids the large error situation of using the II-wPCA method.

**Figure 20:**
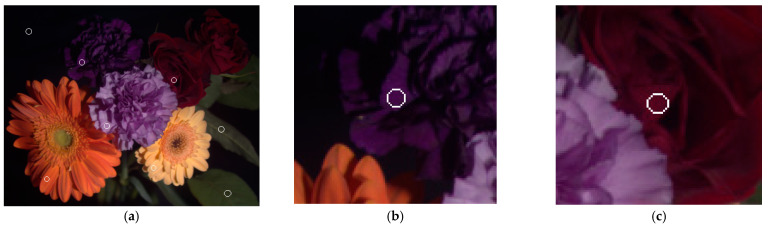

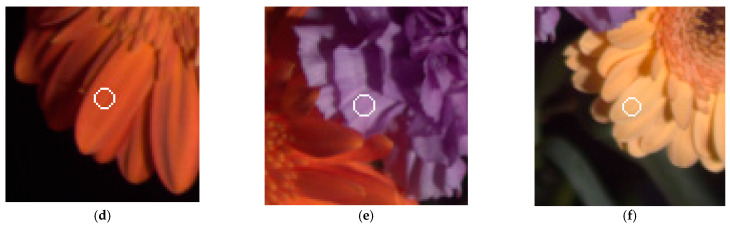
(**a**) Original image of Figure 15d. (**b**–**f**) showing enlarged images on the deep purple, red, orange, purple and yellow flowers in (**a**), respectively. The center of the white circle was an example pixel showing the recovered spectral reflectance, except for the white circle in the upper left corner.

**Figure 21:**
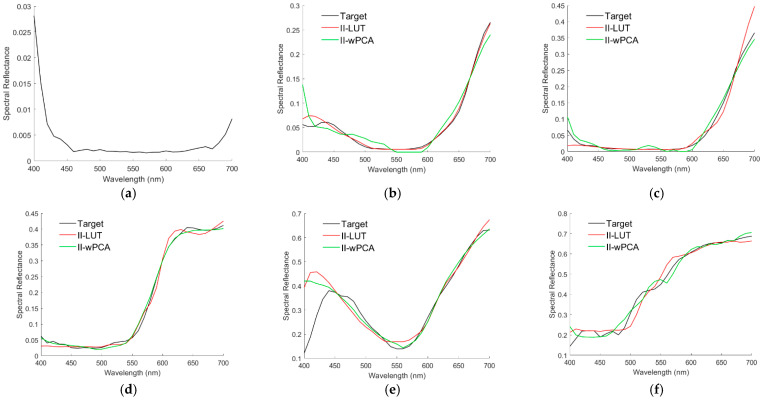
(**a**–**f**) showing the target spectral reflectance of the pixel at the center of the white circle in Figure 20a–f, respectively. (**b**–**f**) also show the recovered spectral reflectance using the II-LUT and II-wPCA methods.

**Figure 22:**
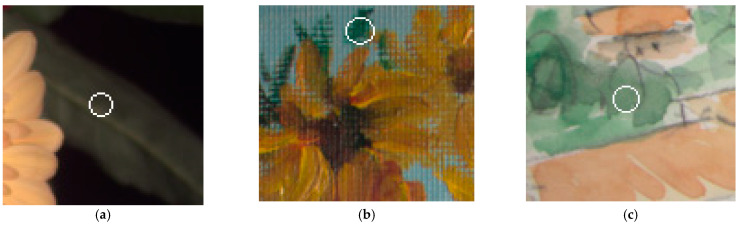

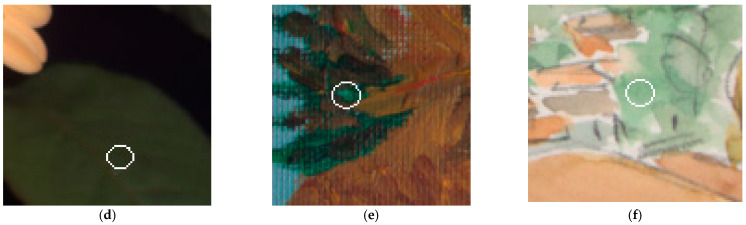
(**a**–**f**) showing enlarged leaf images. (**a**,**d**) are in Figure 20a (“flowers”). (**b**,**e**) are in Figure 15b (“oil painting”). (**c**,**f**) are in Figure 15a (“watercolors”). The center of the white circle was an example pixel showing the recovered spectral reflectance.

Several examples of recovering spectral reflectance from the test images are shown below. Figure 20a is the original image (“flowers”) of Figure 15d without removing nearly black pixels. The white circle in the upper left corner is on the black background. Figure 21a shows the spectral reflectance of the pixel in the center of the white circle. From the spectral reflectance, the black background appears to be a dark purple. However, it will be shown that this spectral reflectance is an error, possibly due to the low SNR as previously described. The centers of other seven white circles in Figure 20a are taken as example pixels. Figure 20b–f show enlarged images of deep purple, red, orange, purple, and yellow flowers, respectively. Enlarged images of the leaf examples at the lower right of Figure 20a are shown in Figure 22a,d.

Figure 21b–f show the target spectral reflectance and recovered spectral reflectance using the II-LUT and II-wPCA methods in the center of the white circles in Figure 20b–f, respectively. The recovered spectral reflectance agrees with the target well, except for the purple example at short wavelengths in Figure 21e. The assessment metric values for these examples are shown in Table 10, where *GFC* values greater than 0.99 are shown in bold. Using the II-LUT method, *GFC* > 0.99 for all examples except the case in Figure 21e. Using the II-wPCA method, *GFC* > 0.99 for all examples except those in Figure 21b,e.

**Figure 23:**
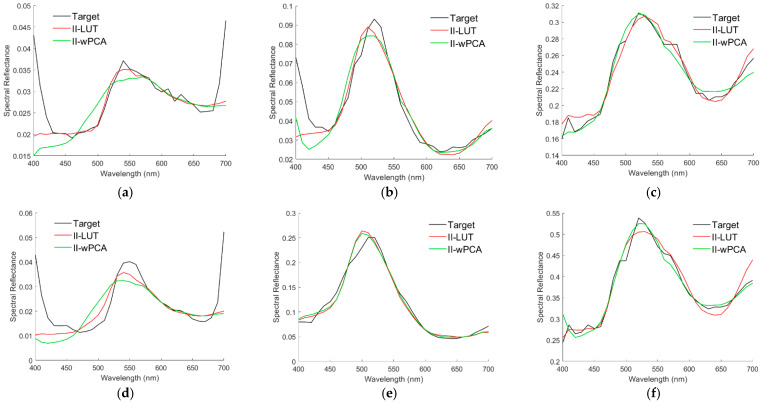
(**a**–**f**) showing the target spectral reflectance of the pixel at the center of the white circle in Figure 22a–f, respectively, where the recovered spectral reflectance using the II-LUT and II-wPCA methods are shown.

Using the II-LUT and II-wPCA methods, Figure 23a,d show the target spectral reflectance and recovered spectral reflectance at the center of the white circles in Figure 22a,d, respectively. Note that the values of target spectral reflectance might be smaller than the actual value because the image values of the CAVE dataset are not calibrated according to the actual irradiance as previously described. Spectral reflectance was not well recovered in both short and long wavelength regions. The spectral reflectance in the 400–460 nm region in Figure 23a,d is almost the same as that of the black background in Figure 21a. Furthermore, the spectral reflectance at 400 nm should be much smaller than that at 550 nm due to the chlorophyll absorption [44]. Therefore, the target spectral reflectance in the short wavelength region in Figure 23a,d is an error. The sharp rise in spectral reflectance near 700 nm is due to the high reflection of leaves in the near IR [44]. This spectral reflectance characteristic is not included in the 202 reference Munsell color chips.

**Figure 24:**
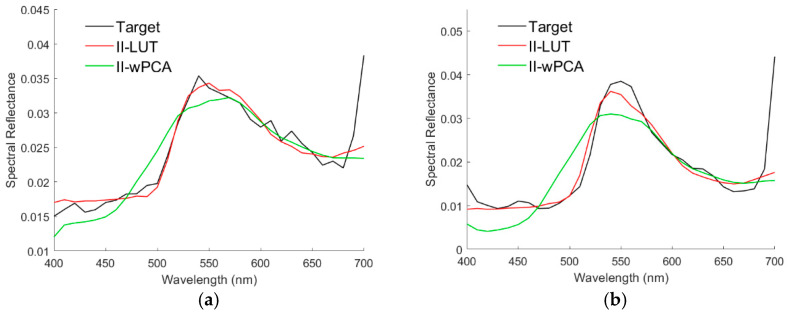
(**a**,**b**) are the Figure 23a,d, respectively, except for the black compensation using the image values of the case in Figure 21a.

It is interesting to compare the spectral reflectance of the leaves in the “flowers”, “oil painting”, and “watercolors” images. Figure 23b,c,e,f show the target spectral reflectance for the center pixel of the white circle in Figure 22b,c,e,f, respectively, where the spectral reflectance recovered using the II-LUT and II-wPCA methods are shown. The recovered spectral reflectance agrees with the target well, except for the short wavelength region in Figure 23b. The spectral reflectance in Figure 23b is less than 0.1. The shape of the spectral reflectance in the 400–460 nm region in Figure 23b is almost the same as that in Figure 21a. This case further validates that the spectral reflectance in the short wavelength region in Figure 21a and Figure 23 a,b,d is erroneous. The spectral reflectance in Figure 23e is larger, and there is no erroneous spectral reflectance in the short wavelength region. Since the pixels corresponding to Figure 21a and Figure 23a,d are from the same test image, black compensation is performed using the image values of the case in Figure 21a to eliminate the erroneous spectral reflectance. The image value of the case in Figure 21a is subtracted from the image values of the cases in Figure 23a,d. Figure 24a,b are the Figure 23a,d, respectively, except for the black compensation.

The assessment metric values of the examples in Figure 23a–f are shown in Table 11, where the values for the black-compensated cases in Figure 24a,b are also shown. *GFC* values greater than 0.99 are shown in bold. Using the II-LUT and II-wPCA methods, *GFC* > 0.99 for the cases in Figure 23c,e,f and Figure 24a. Note that the peak spectral reflectance of leaves is at about 550 nm [44]. Table 12 shows the peak spectral reflectance wavelength for leaves in the “flowers”, “oil painting, “watercolors” images, which also shows the peak wavelengths of the spectral reflectance recovered using the II-LUT and II-wPCA methods. The wavelength resolution of the CAVE image data is 10 nm. As can be seen from Table 12, except for the oil painting leaf in Figure 23e, the error of the predicted peak wavelength using the II-LUT method is less than 10 nm. Except for the real leaf in Figure 23a and Figure 24a and the oil painting leaf in Figure 23e, the error of the predicted peak wavelength using the II-wPCA method is also less than 10 nm. This result might help identify whether the leaves are real or fake from the camera signals.

As the results shown in Figure 21b–f and Figure 24a,b, since spectral reflectance can be recovered well using the 202 reference Munsell color chips, a more accurate spectral reflectance recovery can be achieved using another set of reference samples suitable for vegetation. The use of the II-LUT method and multicolor cameras to evaluate vegetation properties is promising.

## 5. Conclusions

The reconstruction of the spectrum from camera signals was numerically investigated. Conventional LUT method interpolates the spectrum in the signal space. This is an irradiance-dependent LUT (ID-LUT) method because the shape of the reconstructed spectrum shape depends on the sample irradiance. An irradiance-independent LUT (II-LUT) method was proposed, which interpolates the shape and luminance of the reconstructed spectrum in the normalized signal space. The application of this method to recover the surface spectral reflectance using a camera was numerically investigated. The Nikon D5100 and RGBF cameras were taken as the tricolor and quadcolor camera examples, respectively. Munsell color chips were taken as reflective surface examples, where 202 and 1066 color chips were used to prepare reference and test samples, respectively, under D65 illuminant. The results are summarized below.

1. RGBF Camera:

For the RGBF camera, reconstructing the spectra of test samples using the II-LUT method uses one less reference sample than the ID-LUT method. If the irradiance of the test and reference samples is the same, the mean reconstruction error using the II-LUT method was larger than that of the ID-LUT method. Compared to the ID-LUT method, the mean spectrum reconstruction error of the test samples using the II-LUT method was increased by 4.2%. Considering that the irradiance variation will introduce additional error, the mean spectrum reconstruction error using the II-LUT method could be smaller than the ID-LUT method in practice. Therefore, it is better to use the II-LUT method, which can not only reduce the spectrum reconstruction error, but also achieve irradiance independence. In addition to the advantage of irradiance independence, the computation speed using the II-LUT method is much faster than the ID-LUT method. For the case of using the RGBF camera, the ID-LUT and II-LUT methods interpolate the spectrum in 4D signal space and 3D normalized signal space, respectively. To interpolate the considered examples, locating a tetrahedron in 3D space is computationally two orders of magnitude faster than locating a simplex in 4D space.

2. D5100 Camera:

For the D5100 camera, the mean spectrum reconstruction error of test samples using the II-LUT method was increased by 36.3% compared to the ID-LUT method. The increase in mean error is significant because the number of reference samples used to reconstruct the spectrum of the test sample is only 3, which was not enough to reconstruct the spectra well. For the case of using the RGBF camera, the number of reference samples used to reconstruct the spectrum of the test sample is 4, and the reconstruction error was significantly reduced. Using the II-LUT method, the mean spectrum reconstruction error of the test samples reconstructed with the RGBF camera was 48.01% lower than that of the D5100 camera.

3. Comparison of the II-LUT and II-wPCA methods:

Compared to the irradiance independent wPCA (II-wPCA) method, the mean spectrum reconstruction errors using the II-LUT method were reduced by 3.84% and 28.0% with the D5100 and RGBF cameras, respectively. Using the II-wPCA method, the spectrum reconstruction error may further increase due to the estimation errors of the camera spectral sensitivities in field applications [45,46]. Another disadvantage of using the II-wPCA method is the much slower calculation speed compared to the II-LUT method, since the basis spectra need to be derived for each test sample.

4. Irradiance independent color device model (II-CDM):

The II-LUT method can be easily modified to an irradiance-independent color device model (II-CDM). Conventional II-CDM is based on root polynomial regression. The II-CDM based on the II-LUT method was found to be more accurate than the root polynomial regression-based II-CDM for the examples considered but required the measurement of auxiliary reference samples. The application of the II-LUT method in II-CDM requires further study.

5. Reconstruction of spectral reflectance images

The selection of reference/training samples is crucial for the spectrum reconstruction. Although the reference samples were not selected for the considered test images, the mean spectrum reconstruction error using the II-LUT method was low or moderate for most image pixels, except for some special cases. Reflected glare should be avoided when capturing images to recover spectral reflectance.

Using the conventional tricolor camera and the ID-LUT method for spectrum reconstruction has the advantages of no need to measure/estimate camera spectral sensitivity functions, fast detection speed, high spatial resolution, and low cost. Using the conventional tricolor camera and the II-LUT method has the additional advantage of irradiance independence, but the spectrum reconstruction error increases significantly. The spectrum reconstruction error can be effectively reduced using the quadcolor camera, where its color filter array is compatible with the conventional tricolor camera in demosaicing. For the examples considered, using the RGBF camera and the II-LUT method has the additional advantages of irradiance independence and fast computation speed, while the spectrum reconstruction error was slightly increased compared to using the ID-LUT method.

Although the spectral sensitivities of the two example cameras are based on the D5100 camera except for the F channel of the RGBF camera, the proposed II-LUT method can generally be applied to the cameras with other spectral sensitivity characteristics. Further studies are required to implement the proposed method for field application. One possible implementation of the quadcolor camera is to use the dual cameras built into commercially available smartphones, one of which is a conventional tricolor camera and the other a monochrome camera, such as the Huawei P9 smartphone. The monochrome camera is usually used to enhance image resolution and reduce noise. With the dual cameras, we have the RGB signals from the tricolor camera and the F signal from the monochrome camera, but care must be taken with the pixel alignment of the two cameras.

## Figures and Tables

**Table 1:** Munsell annotation of color chip and the *β_k_* value in Equation (13) for the cases shown in Figure 10a–f. The maximum *β_k_* value is shown in bold. The *β_k_* value corresponding to the normalized ARS neighborhood is indicated with “*”.

	Annotation	*β*_1_	*β*_2_	*β*_3_	*β*_4_
(a)	5Y 8.5/8	**0.465**	0.200	0.047	0.288
(b)	10P 7/8	0.296	**0.498**	0.195	0.011
(c)	2.5R 4/12	0.258 *	0.177	**0.348**	0.217
(d)	2.5G 7/6	0.006 *	**0.452**	0.449	0.093
(e)	10BG 4/8	0.067 *	0.334	**0.587**	0.012
(f)	5PB 4/12	0.066 *	0.242 *	0.260	**0.431**

**Table 2:** The *E*_Ref_ and Δ*E*_00_ values for the cases shown in Figure 11a–f. The best values are shown in bold.

Metric	*E*_Ref_				Δ*E*_00_			
Method	ID-LUT	II-LUT	ID-wPCA	II-wPCA	ID-LUT	II-LUT	ID-wPCA	II-wPCA
(a)	**0.0175**	0.0184	0.0311	0.0297	**0.3824**	0.4387	0.8579	0.4833
(b)	0.0108	0.0193	0.0076	**0.0066**	0.1395	0.1455	0.1045	**0.0904**
(c)	0.0071	**0.0056**	0.0076	0.0144	0.0852	**0.0634**	0.1721	0.2512
(d)	0.0032	**0.0030**	0.0124	0.0062	0.1043	0.0968	0.5428	**0.0377**
(e)	**0.0082**	0.0130	0.0203	0.0212	**0.7238**	1.3306	2.0505	2.1487
(f)	0.0308	**0.0236**	0.0343	0.0347	0.7133	**0.4634**	1.1166	1.3626

**Table 3:** Assessment metric statistics for the test samples using the ID-LUT and II-LUT methods, where the camera is the RGBF camera.

**Metric**	**Method**	**ID-LUT**			**II-LUT**		
	**Sample**	**All**	**Inside**	**Outside**	**All**	**Inside**	**Outside**
	**No.**	1066	726	340	1066	935	131
*E*_Ref_	mean *μ*	0.0089	0.0077	0.0115	0.0093	0.0089	0.0119
	std *σ*	0.0079	0.0064	0.0100	0.0096	0.0094	0.0105
	PC50	0.0064	0.0055	0.0082	0.0060	0.0057	0.0084
	PC98	0.0345	0.0262	0.0416	0.0429	0.0428	0.0475
	MAX	0.0567	0.0451	0.0567	0.0733	0.0733	0.0608
*GFC*	mean *μ*	0.9989	0.9993	0.9980	0.9988	0.9989	0.9979
	std *σ*	0.0023	0.0012	0.0035	0.0034	0.0033	0.0041
	PC50	0.9996	0.9997	0.9992	0.9996	0.9997	0.9994
	MIN	0.9669	0.9832	0.9669	0.9387	0.9387	0.9697
	RGF99	0.9887	0.9972	0.9706	0.9831	0.9840	0.9771
Δ*E*_00_	mean *μ*	0.3992	0.3865	0.4263	0.3062	0.2860	0.4500
	std *σ*	0.3590	0.3535	0.3696	0.3669	0.3456	0.4700
	PC50	0.2792	0.2734	0.2902	0.1899	0.1756	0.2760
	PC98	1.4447	1.4441	1.4453	1.5388	1.4095	1.8562
	MAX	1.8843	1.8843	1.8099	3.3855	3.3855	2.7694
*SCI*	mean *μ*	3.5296	2.9027	4.8682	3.0399	2.7015	5.4546
	std *σ*	2.7643	2.0677	3.4962	3.3538	2.9824	4.6412
	PC50	2.7585	2.3611	3.9021	1.9979	1.8447	3.7871
	PC98	11.9210	9.1115	16.2671	16.3741	11.5673	20.1699
	MAX	20.0506	14.5365	20.0506	27.5602	24.5418	27.5602

**Table 4:** Assessment metric statistics for the test samples using the ID-wPCA and II-wPCA methods, where the camera is RGBF camera.

**Metric**	**Method**	**ID-wPCA**			**II-wPCA**		
	**Sample**	**All**	**Inside**	**Outside**	**All**	**Inside**	**Outside**
	**No.**	1066	726	340	1066	935	131
*E*_Ref_	mean *μ*	0.0095	0.0080	0.0127	0.0129	0.0119	0.0200
	std *σ*	0.0087	0.0068	0.0110	0.0282	0.0266	0.0370
	PC50	0.0066	0.0059	0.0091	0.0072	0.0066	0.0117
	PC98	0.0338	0.0288	0.0490	0.0729	0.0623	0.0949
	MAX	0.0742	0.0742	0.0738	0.6106	0.6106	0.3473
*GFC*	mean *μ*	0.9985	0.9992	0.9972	0.9970	0.9976	0.9930
	std *σ*	0.0046	0.0029	0.0068	0.0173	0.0156	0.0260
	PC50	0.9995	0.9996	0.9990	0.9995	0.9996	0.9987
	MIN	0.9315	0.9364	0.9315	0.6553	0.6553	0.7247
	RGF99	0.9765	0.9945	0.9382	0.9503	0.9636	0.8550
Δ*E*_00_	mean *μ*	0.4257	0.3475	0.5926	0.4598	0.4172	0.7639
	std *σ*	0.4558	0.3213	0.6252	0.9449	0.9473	0.8722
	PC50	0.2942	0.2689	0.3985	0.2821	0.2678	0.4660
	PC98	1.7690	1.1557	2.6548	2.2236	1.8631	2.9892
	MAX	5.0328	3.3353	5.0328	22.6299	22.6299	6.1797
*SCI*	mean *μ*	3.8938	2.9955	5.8118	4.3291	3.7447	8.4998
	std *σ*	4.2229	2.9221	5.6872	7.9785	7.1530	11.5717
	PC50	2.6196	2.2980	4.0662	2.4480	2.3014	5.1812
	PC98	15.9216	9.1608	19.4698	19.4483	16.2815	40.9452
	MAX	46.2716	42.5477	46.2716	148.25	148.25	99.10

**Table 5:** Assessment metric statistics for the test samples using the ID-LUT and II-LUT methods, where the camera is the D5100 camera.

**Metric**	**Method**	**ID-LUT**			**II-LUT**		
	**Sample**	**All**	**Inside**	**Outside**	**All**	**Inside**	**Outside**
	**No.**	1066	864	202	1066	1003	63
*E*_Ref_	mean *μ*	0.0131	0.0120	0.0180	0.0179	0.0170	0.0314
	std *σ*	0.0124	0.0107	0.0169	0.0222	0.0212	0.0315
	PC50	0.0089	0.0087	0.0125	0.0113	0.0108	0.0193
	PC98	0.0540	0.0485	0.0698	0.0771	0.0711	0.1290
	MAX	0.1038	0.0859	0.1038	0.2399	0.2399	0.1347
*GFC*	mean *μ*	0.9971	0.9974	0.9961	0.9962	0.9963	0.9947
	std *σ*	0.0074	0.0071	0.0085	0.0100	0.0102	0.0053
	PC50	0.9993	0.9994	0.9985	0.9989	0.9990	0.9960
	MIN	0.9000	0.9000	0.9325	0.8606	0.8606	0.9767
	RGF99	0.9343	0.9375	0.9208	0.9212	0.9262	0.8413
Δ*E*_00_	mean *μ*	0.4215	0.4239	0.4111	0.4778	0.4761	0.5056
	std *σ*	0.4065	0.4182	0.3529	0.5218	0.5237	0.4935
	PC50	0.2796	0.2795	0.2830	0.2997	0.2997	0.3155
	PC98	1.6478	1.6900	1.4386	2.0989	2.1033	1.9165
	MAX	2.5918	2.5918	1.8207	3.8500	3.8500	1.9526
*SCI*	mean *μ*	4.1253	3.7503	5.7291	4.5919	4.3701	8.1223
	std *σ*	3.2381	2.9266	3.9487	4.3776	4.1878	5.6820
	PC50	3.1484	2.9310	4.7827	3.0672	2.9512	7.0131
	PC98	13.7172	12.1239	16.4027	18.5869	18.0068	22.1290
	MAX	25.3596	25.2299	25.3596	28.2956	28.2956	23.5022

**Table 6:** Assessment metric statistics for the test samples using the ID-wPCA and II-wPCA methods, where the camera is the D5100 camera.

**Metric**	**Method**	**ID-wPCA**			**II-wPCA**		
	**Sample**	**All**	**Inside**	**Outside**	**All**	**Inside**	**Outside**
	**No.**	1066	864	202	1066	1003	63
*E*_Ref_	mean *μ*	0.0121	0.0110	0.0169	0.0186	0.0164	0.0527
	std *σ*	0.0121	0.0098	0.0181	0.0437	0.0339	0.1139
	PC50	0.0086	0.0082	0.0115	0.0101	0.0097	0.0282
	PC98	0.0531	0.0437	0.0783	0.0898	0.0816	0.3358
	MAX	0.1152	0.0817	0.1152	0.9062	0.8931	0.9062
*GFC*	mean *μ*	0.9978	0.9984	0.9950	0.9940	0.9948	0.9806
	std *σ*	0.0056	0.0032	0.0108	0.0258	0.0221	0.0575
	PC50	0.9994	0.9994	0.9988	0.9991	0.9992	0.9942
	MIN	0.9017	0.9618	0.9017	0.5698	0.6142	0.5698
	RGF99	0.9493	0.9676	0.8713	0.9015	0.9182	0.6349
Δ*E*_00_	mean *μ*	0.3884	0.3443	0.5769	0.6517	0.5890	1.6495
	std *σ*	0.4133	0.3235	0.6416	1.6133	1.4047	3.4273
	PC50	0.2573	0.2422	0.3259	0.3208	0.3057	0.7662
	PC98	1.8615	1.3261	2.5734	3.0596	2.7772	14.4566
	MAX	2.8330	2.5378	2.8330	34.4832	34.4832	24.8190
*SCI*	mean *μ*	3.8000	3.1831	6.4384	5.6905	4.9779	17.0352
	std *σ*	3.9295	2.7777	6.3288	11.4821	9.6707	24.7828
	PC50	2.6841	2.5232	4.3897	3.1649	2.9897	10.4229
	PC98	16.7371	11.2917	29.0811	29.5441	23.2148	97.8808
	MAX	34.6758	28.8125	34.6758	242.57	242.57	183.37

**Table 7:** Color difference Δ*E*_00_ statistics for the test samples using the third-order RPRM, where the cases with the D5100 and RGBF cameras are shown.

**Metric**	**Camera**	**D5100**			**RGBF**		
	**Sample**	**All**	**Inside**	**Outside**	**All**	**Inside**	**Outside**
	**No.**	1066	1003	63	1066	935	131
Δ*E*_00_	mean *μ*	0.5140	0.4921	0.8630	0.4160	0.3671	0.7652
	std *σ*	0.4635	0.4425	0.6286	0.5363	0.3285	1.2001
	PC50	0.4005	0.3843	0.6918	0.3020	0.2935	0.4018
	PC98	2.0504	1.9743	2.7162	1.7563	1.2252	5.4271
	MAX	3.6353	3.6353	2.8645	8.9801	3.3099	8.9801

**Table 8:** Assessment metric statistics for the test images in Figure 15a–f using the II-LUT method and RGBF camera.

Metric	Image	Watercolors	Oil Painting	Egyptian Statue	Flowers	Cloth	Face
	Pixel No.	181,125	178,161	50,884	93,132	229,439	55,571
*E*_Ref_	mean *μ*	**0.0185**	0.0154	0.0090	0.0188	**0.0237**	0.0245
	std *σ*	0.0071	0.0084	0.0059	0.0152	0.0070	0.0129
	PC50	0.0176	0.0125	0.0075	0.0133	0.0240	0.0239
	PC98	0.0336	0.0417	0.0227	0.0680	0.0378	0.0503
	MAX	0.0840	0.0821	0.0869	0.1052	0.0714	0.0814
*GFC*	mean *μ*	**0.9988**	0.9928	0.9888	**0.9835**	**0.9894**	0.9937
	std *σ*	0.0013	0.0110	0.0121	0.0209	0.0099	0.0023
	PC50	0.9991	0.9955	0.9925	0.9922	0.9917	0.9939
	MIN	0.9308	0.7624	0.5784	0.6481	0.8625	0.9347
	RGF99	0.9969	0.8590	0.6646	0.5345	0.5881	0.9502
Δ*E*_00_	mean *μ*	**0.6184**	0.6179	0.7256	**0.8189**	**0.7777**	1.8170
	std *σ*	0.4407	0.4063	0.2119	0.4279	0.4252	0.4633
	PC50	0.5056	0.4928	0.7170	0.7568	0.8392	1.9012
	PC98	1.7259	1.7173	1.2183	1.9411	1.7505	2.5505
	MAX	2.8756	4.1422	3.8495	3.4148	3.7200	3.5040
*SCI*	mean *μ*	**4.9076**	6.7761	8.5796	**9.1071**	**9.0849**	11.3164
	std *σ*	1.8871	2.4782	3.0685	3.2774	3.3666	3.1826
	PC50	4.4999	6.2873	7.8708	8.6641	8.5609	11.5339
	PC98	9.2387	14.1738	15.2429	17.2996	15.0797	17.1621
	MAX	17.7182	30.2017	29.4085	28.9355	21.0533	22.1692

**Table 9:** Assessment metric statistics for the test images in Figure 15a–f, using the II-wPCA method and RGBF camera.

Metric	Image	Watercolors	Oil Painting	Egyptian Statue	Flowers	Cloth	Face
	Pixel No.	181,125	178,161	50,884	93,132	229,439	55,571
*E*_Ref_	mean *μ*	0.0414	**0.0146**	**0.0076**	**0.0169**	0.0252	**0.0170**
	std *σ*	2.8076	0.0500	0.0111	0.0149	0.0193	0.0132
	PC50	0.0189	0.0119	0.0065	0.0113	0.0231	0.0158
	PC98	0.0932	0.0369	0.0170	0.0620	0.0547	0.0517
	MAX	1146.11	17.4630	1.4987	0.3757	6.4953	1.2401
*GFC*	mean *μ*	0.9960	0.9928	**0.9892**	0.9820	0.9875	**0.9969**
	std *σ*	0.0215	0.0143	0.0169	0.0271	0.0218	0.0040
	PC50	0.9990	0.9964	0.9956	0.9959	0.9926	0.9976
	MIN	0.2884	0.3860	0.3507	0.6628	0.2455	0.4737
	RGF99	0.9456	0.8549	0.7571	0.5911	0.7036	0.9836
Δ*E*_00_	mean *μ*	0.9505	**0.5816**	**0.7077**	1.0565	1.1349	**1.1082**
	std *σ*	1.7508	0.6207	0.5520	1.4009	1.2913	0.6709
	PC50	0.6672	0.4579	0.6019	0.7191	0.6841	0.9997
	PC98	2.8982	1.7340	1.3723	5.0667	5.0151	2.6072
	MAX	125.390	66.5353	59.0912	29.8370	84.2292	54.3326
*SCI*	mean *μ*	6.8898	**6.0605**	**7.7003**	10.4127	11.4622	**8.6193**
	std *σ*	48.1646	6.4039	4.3459	6.4523	7.0328	4.5566
	PC50	4.6353	5.1531	7.5687	8.6493	11.3655	8.3811
	PC98	16.9898	15.6412	11.7063	29.8017	22.4230	17.7990
	MAX	14596.99	1238.83	528.81	282.87	1258.84	574.03

**Table 10:** Assessment metric values for the cases in Figure 21b–f. *GFC* values greater than 0.99 are shown in bold.

Method	II-LUT			II-wPCA		
Figure	(a)	(b)	(c)	(a)	(b)	(c)
*E*_Ref_	NA	0.0067	0.0221	NA	0.0195	0.0127
*GFC*	NA	**0.9972**	**0.9900**	NA	0.9765	**0.9949**
Δ*E*_00_	NA	0.1433	0.7499	NA	2.6129	0.8046
*SCI*	NA	5.5609	7.0299	NA	24.1724	19.4611
Figure	(d)	(e)	(f)	(d)	(e)	(f)
*E*_Ref_	0.0138	0.0808	0.0303	0.0081	0.0743	0.0287
*GFC*	**0.9983**	0.9781	**0.9981**	**0.9994**	0.9807	**0.9983**
Δ*E*_00_	0.3974	1.0014	0.9931	0.4924	0.7027	0.6500
*SCI*	9.0932	13.0039	7.8948	7.1192	8.5063	8.3355

**Table 11:** Assessment metric values for the cases in Figure 23a–f. Values for the black-compensated cases in Figure 24a,b are also shown, where the data column is denoted by “*”. *GFC* values greater than 0.99 are shown in bold.

Method	II-LUT				II-wPCA			
Figure	23a	24a *	23b	23c	23a	24a	23b	23c
*E*_Ref_	0.0060	0.0027	0.0097	0.0083	0.0072	0.0033	0.0094	0.0080
*GFC*	0.9806	**0.9946**	0.9821	**0.9994**	0.9711	**0.9916**	0.9831	**0.9994**
Δ*E*_00_	0.3368	0.1942	0.6119	0.3405	1.0111	0.6331	0.8657	0.2020
*SCI*	2.0894	2.1451	5.6005	3.0445	4.8180	4.0099	6.8681	2.4910
Figure	23d	24b *	23e	23f	23d	24b	23e	23f
*E*_Ref_	0.0092	0.0052	0.0113	0.0172	0.0105	0.0071	0.0104	0.0170
*GFC*	0.9356	0.9723	**0.9966**	**0.9990**	0.9135	0.9466	**0.9971**	**0.9990**
Δ*E*_00_	1.3769	0.5505	0.7517	0.1674	2.2002	1.8859	0.5601	0.2436
*SCI*	7.6201	4.1116	6.6695	4.2577	12.4951	12.1565	5.8404	3.6242

**Table 12:** Peak spectral reflectance wavelengths of leaves in the “flowers”, “oil painting, “watercolors” images, where the corresponding figures are indicated. Values for the black-compensated cases in Figure 24a,b are also shown, where the data column is denoted by “*”. Wavelengths are in unit of nm. Using the II-LUT and II-wPCA methods, predicted peak wavelengths with errors greater than 10 nm are shown in bold.

Image	Flowers				Oil Painting		Watercolors	
Figure	23a	24a *	23d	24b *	23b	23e	23c	23f
Target	540	540	550	550	520	520	520	520
II-LUT	540	550	540	540	510	**500**	530	530
II-wPCA	**570**	**570**	540	540	520	**500**	520	520

## Data Availability

1. Spectral sensitivities of the Nikon D5100 camera are available: http://spectralestimation.wordpress.com/data/. 2. Spectral reflectance of matt Munsell color chips are available: https://sites.uef.fi/spectral/munsell-colors-matt-spectrofotometer-measured/. 3. Spectral transmittance of the UV/IR cut filter is available: https://agenaastro.com/downloads/manuals/baader-uvir-cut-filter-stat-sheet.pdf. 4. CAVE multispectral dataset is available: https://www1.cs.columbia.edu/CAVE/databases/multispectral/. All are accessed on 21 October 2022.

## Abbreviations

**Abbreviation**	**Definition**
ARS	Auxiliary Reference Sample
CDM	Color Device Model
CFA	Color Filter Array
CMF	Color-Matching Function
GFC	Goodness-of-Fit Coefficient
ID-LUT	Irradiance-Dependent Look-Up Table
ID-wPCA	Irradiance-Dependent Weighted Principal Component Analysis
II-CDM	Irradiance-Independent Color Device Model
II-LUT	Irradiance-Independent Look-Up Table
II-LUT-CDM	Irradiance-Independent Look-Up Table Color Device Model
II-wPCA	Irradiance-Independent weighted Principal Component Analysis
LUT	Look-Up Table
MAX	Maximum
MIN	Minimum
PC50	50th Percentile
PC98	98th Percentile
PCA	Principal Component Analysis
RGB	Red, Green, and Blue
RGBF	Red, Green, Blue, and Free
RGF99	Ratio of Good Fit (the ratio of samples with *GFC* > 0.99)
RMS	Root Mean Square
RPRM	Root Polynomial Regression Model
SCI	Spectral Comparison Index
SNR	Signal-to-Noise Ratio
wPCA	Weighted Principal Component Analysis
